# Targeting macrophages in hematological malignancies: recent advances and future directions

**DOI:** 10.1186/s13045-022-01328-x

**Published:** 2022-08-17

**Authors:** Wei Li, Fang Wang, Rongqun Guo, Zhilei Bian, Yongping Song

**Affiliations:** Department of Hematology, The First Affiliated Hospital of Zhengzhou University, Zhengzhou, 450052 Henan China

**Keywords:** Macrophage, CD47, SIRPα, PD-1/PD-L1, CD24/SIGLEC-10, MHC-I/LILRB1/2, CSF1R inhibitor, BsAbs, MARCO, TLR agonist, Tim-4, CAR-M

## Abstract

Emerging evidence indicates that the detection and clearance of cancer cells via phagocytosis induced by innate immune checkpoints play significant roles in tumor-mediated immune escape. The most well-described innate immune checkpoints are the “don’t eat me” signals, including the CD47/signal regulatory protein α axis (SIRPα), PD-1/PD-L1 axis, CD24/SIGLEC-10 axis, and MHC-I/LILRB1 axis. Molecules have been developed to block these pathways and enhance the phagocytic activity against tumors. Several clinical studies have investigated the safety and efficacy of CD47 blockades, either alone or in combination with existing therapy in hematological malignancies, including myelodysplastic syndrome (MDS), acute myeloid leukemia (AML), and lymphoma. However, only a minority of patients have significant responses to these treatments alone. Combining CD47 blockades with other treatment modalities are in clinical studies, with early results suggesting a synergistic therapeutic effect. Targeting macrophages with bispecific antibodies are being explored in blood cancer therapy. Furthermore, reprogramming of pro-tumor macrophages to anti-tumor macrophages, and CAR macrophages (CAR-M) demonstrate anti-tumor activities. In this review, we elucidated distinct types of macrophage-targeted strategies in hematological malignancies, from preclinical experiments to clinical trials, and outlined potential therapeutic approaches being developed.

## Background

**Fig. 1:**
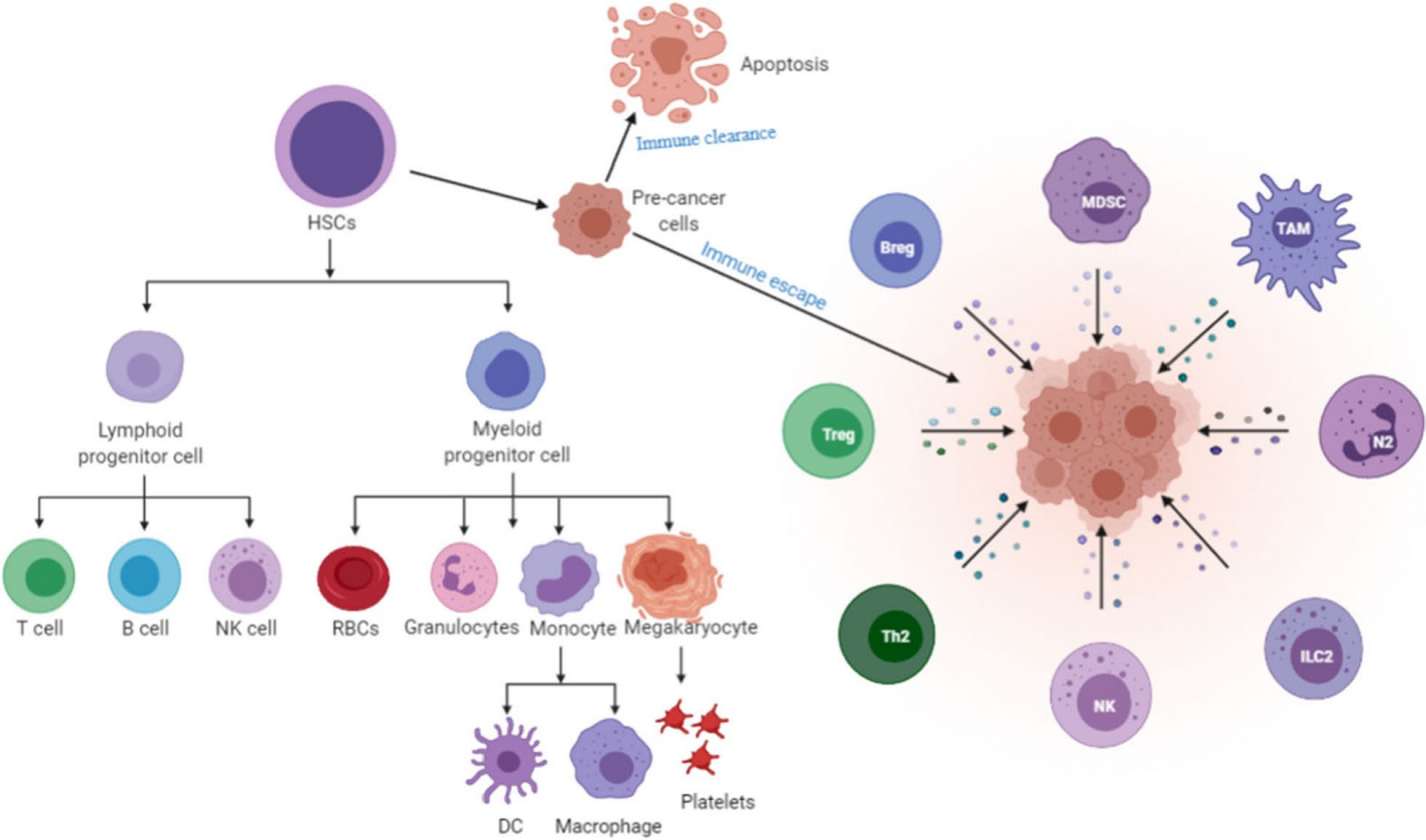
An overview of normal hematopoiesis and possible immune escape mechanisms for blood cancers: normally, hematopoietic stem cells (HSCs) develop, in a fate-determined manner, into spectrum-specific hematopoietic progenitor cells, which then differentiate into relative terminal cells. The terminal cells maintain stable hematopoietic development. Inevitably, some precancerous cells appear during the development of the hematopoietic system, but are normally cleared by the immune system. However, precancerous cells can develop into various kinds of hematological malignancies when the immune system is compromised by T cells, B cells, NK cells, MDSC, TAM, and so on

Both CTLA-4 and PD-1/PD-L1 blockades have demonstrated impressive, durable anti-tumor responses [1–7]. However, only a minority of patients achieve maximal benefit from monotherapy, most likely due to the highly heterogeneous and complex immune cancer microenvironment [8–11]. Therefore, investigations into combination strategies, new checkpoints, and checkpoint inhibitors are underway [12–16]. Various factors regulate hematopoiesis to maintain normal blood cell production [17]. However, precancerous cells can also be generated, which can either undergo apoptosis and be cleared by the immune system or develop into hematological malignancies following immune evasions [18, 19] (Fig. 1). Several immune evasion mechanisms beyond the suppression of anticancer T cell responses have been reported in previous studies [19–27]. Tumor-associated macrophages (TAMs), a specific subpopulation of macrophages, represent a large fraction of infiltrating immune cells within the tumor microenvironment (TME) in human cancers [28, 29]. TAMs are considered a potentially effective therapeutic target since they drive tumor progression, metastasis, and recurrence via multiple mechanisms [28, 29].

The first macrophage-targeted therapeutic agent is the CD47 monoclonal antibody (mAb) [30]. In the late 2000s, the cross talk of CD47/SIRPα was recognized as the first checkpoint associated with tumor phagocytosis (also known as a “don’t eat me” signal) [31]. CD47 expression has been found to be considerably elevated in numerous hematological malignancies, as well as solid cancers [32, 33]. Additionally, a significant positive correlation has been reported between high levels of CD47 expression and a poor prognosis in cancers [34–36]. Blocking the CD47/SIRPα cross talk has been shown to enhance anti-tumor activities. Therefore, blocking the CD47/SIRPα cross talk may be a promising approach for cancer immunotherapy either on its own or via integration with other tumor-targeted therapies, as reported in numerous preclinical studies [35, 37–40]. Clinical studies confirm the importance of inhibiting the CD47/SIRPα interaction in various hematological malignancies, including myelodysplastic syndrome (MDS), acute myeloid leukemia (AML) [41], and relapsed/refractory non-Hodgkin’s lymphoma (R/R-NHL) [42]. Furthermore, other “don’t eat me” signals, including the PD-1/PD-L1 axis [43], MHC-I/LILRB1/2 axis [44–46], and CD24/SIGLEC-10 axis [47], have been reported to modulate anticancer innate immune responses via macrophage-mediated phagocytosis. Macrophage depletion through inhibition of the CSF1/CSF1R suppresses the differentiation, proliferation, and survival of murine M2 macrophages [48]. Additionally, blocking the CSF1/CSF1R axis can functionally repolarize macrophages toward M1 macrophages, enhance the role of macrophages in antigen presentation, and increase anti-tumor T cell responses [49]. Despite the findings of these preclinical studies, monotherapy with macrophage-targeted therapeutics has demonstrated high rates of adverse effects and relatively lower clinical responses. Thus, other strategies should be developed to improve these shortcomings.

Bispecific antibodies (BsAbs) can recognize and bind two diverse antigens or epitopes to promote treatment efficacy and reduce the risk of adverse events [50–52]. Accordingly, CD47-targeted BsAbs may be a promising strategy to overcome limitations with CD47 blockades and further improve therapeutic efficacy for hematological malignancies. Indeed, CD47/CD20 [53–55], CD47/CD19 [56–59], CD47/CD33 [60], CD47/PD-L1 [61], and CD47/PD-1 [62] show selective CD47 blocking in an antigen-dependent manner in preclinical studies.

The reprogramming of pro-tumor macrophages (M1) to anti-tumor macrophages (M2) has shown potential application as cancer therapeutics [63, 64]. Recently, preclinical and clinical studies have assessed several such therapeutic approaches, including macrophage receptors with collagenous structure (MARCO), toll-like receptors (TLRs) agonists, and T cell immunoglobulin and mucin domain containing 4 (Tim-4) blockades [64–68]. More recent studies have focused on chimeric antigen receptor macrophages (CAR-Ms). These studies have demonstrated effective anti-tumor responses in solid tumors and hematological malignancies in an antigen-specific manner [65–68].

This review provides an overview of current knowledge of macrophage-targeted therapeutics in preclinical and clinical research. Moreover, we outline these therapies for hematological malignancies.

1. **Checkpoints for macrophage-mediated phagocytosis**

**Fig. 2:**
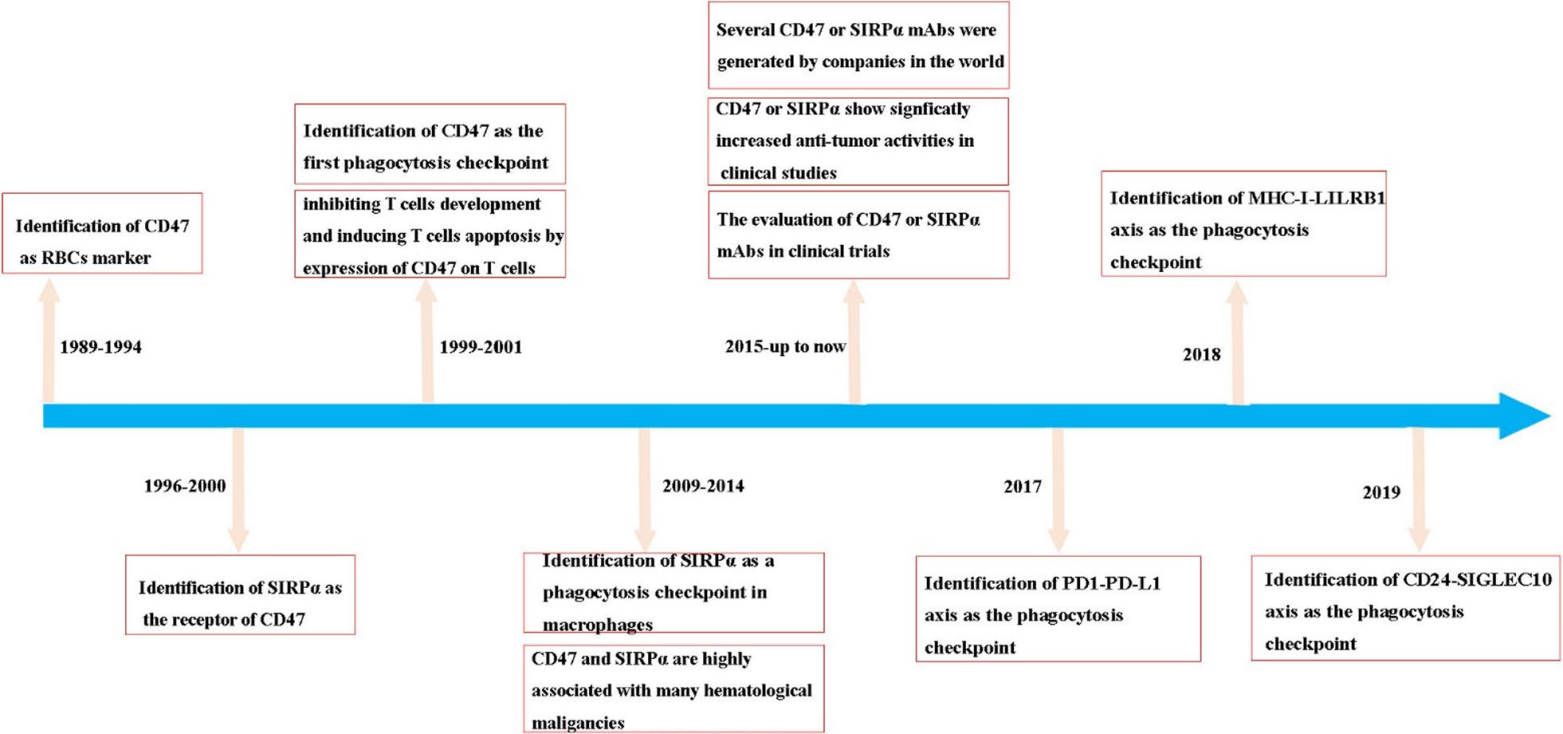
History of discovering phagocytosis-associated checkpoint inhibitors: over the past two decades, a great deal of progress has been made in identifying phagocytosis-associated checkpoints

Since the discovery of the first tumor phagocytosis-related checkpoint, namely the CD47/SIRPα axis, in the late 2000s, other tumor phagocytosis-related checkpoints have been identified: the PD-1/PD-L1 axis, MHC-I/LILRB1 axis, and CD24/SIGLEC-10 axis (Fig. 2). Then, a variety of mAbs or fusion proteins were produced against these four different macrophage phagocytosis-related checkpoints. And the preliminary clinical efficacy and TEAEs of some CD47 mAbs or fusion proteins have been reported.

### CD47/SIRPα axis

#### The essential function of the CD47/SIRPα interaction

CD47 was first established as a membrane protein expressed on normal red blood cells (RBCs) [69]. Previous research has shown that splenic red pulp macrophages (RPMs), liver tissue-resident macrophages (TRMs), and bone marrow erythroblastic island (EBI) macrophages quickly remove senescent RBCs with diminished CD47 expression [70–74]. However, CD47 expression on normal erythroid cells avoids clearance by binding to the macrophage inhibitory receptor SIRPα [75]. Evidence accumulated in recent years suggests that SIRPα is a membrane protein classified as a member of the immunoglobulin superfamily, primarily expressed by myeloid cells such as macrophages and dendritic cells (DCs) [30]. By further elucidating the mechanism of phagocytosis inhibition, researchers have reported that macrophages’ SIRPα interacts with CD47 expressed on neighboring cells, resulting in phosphorylation of the SIRPα cytoplasmic immunoreceptor tyrosine-based inhibition motif. This process results in the recruitment of SHP-1 and SHP-2 phosphatases [76]. The downstream signaling cascade prevents myosin-IIA aggregation at the phagocytic synapse, resulting in phagocytic inhibition [76]. Thus, the CD47/SIRPα axis is mainly regarded as a “don’t eat me” signal, allowing CD47-expressing cells to evade macrophage-mediated phagocytosis [76]. Indeed, previous investigations using mouse models have exhibited that wild-type macrophages rapidly eliminate CD47^−/−^ cells [77]. Studies have also indicated that most cell types, such as erythroblasts, platelets, hematopoietic stem cells (HSCs) [75, 78–80], elevate CD47 expression on their surfaces to evade phagocytosis by macrophages. A similar mechanism preventing phagocytosis by macrophages has been reported for nearly all tumor types, including AML, NHL, and MDS [33, 81]. Collectively, these results confirm that CD47/SIRPα cross talk functions as a negative phagocytosis-associated immune checkpoint.

(2). **The** **anti-tumor mechanisms of blocking** **CD47/SIRPα cross talk**

**Fig. 3:**
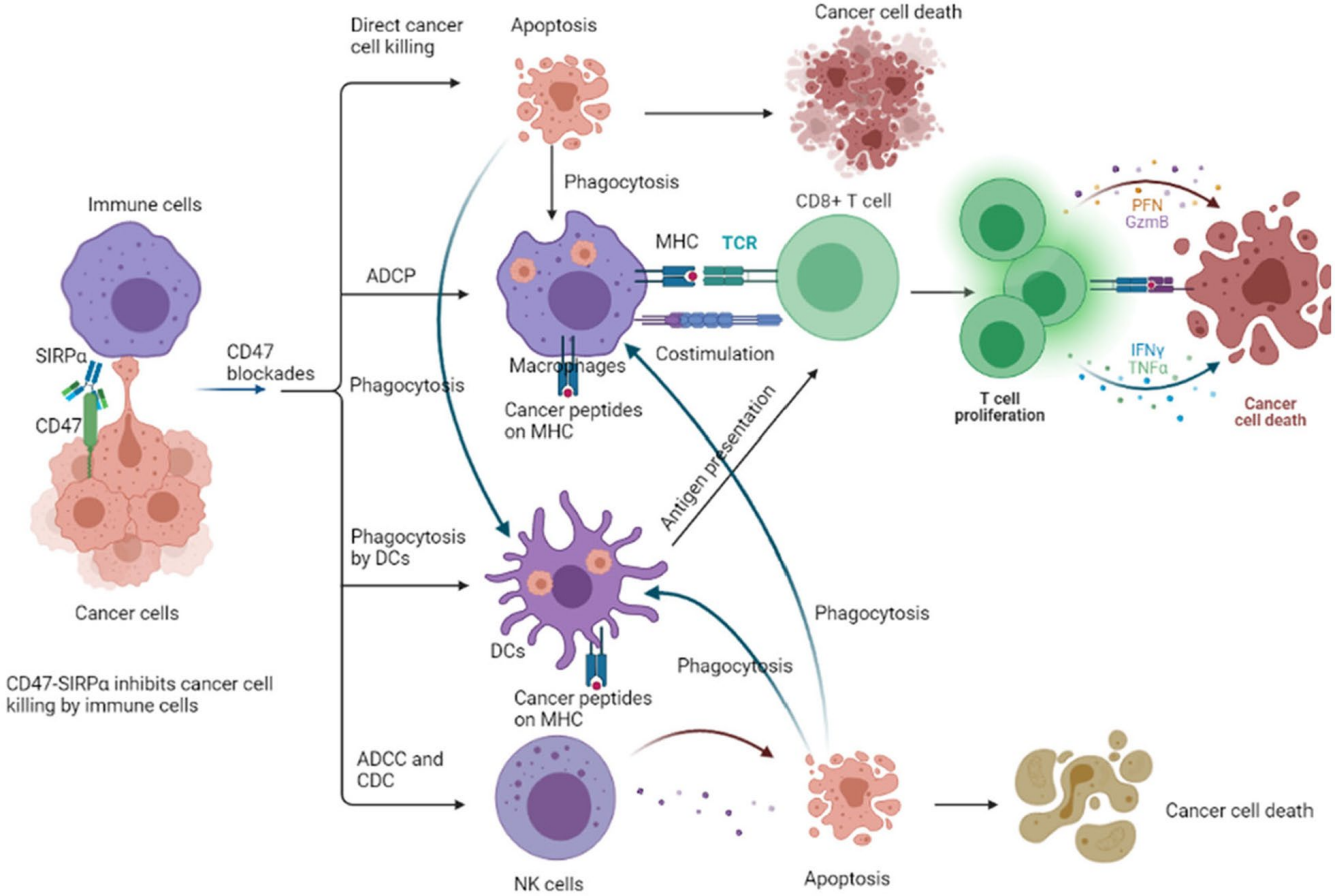
Anti-tumor mechanisms of blocking CD47/SIRPα interaction: through blocking the CD47/SIRPα interaction, anti-tumor effects were induced via direct cancer-killing effects, antibody-dependent cellular phagocytosis (ADCP), antigen presentation and T cell immune responses, antibody-dependent cytotoxicity (ADCC), and complement-dependent cytotoxicity (CDC)

Several investigations have exhibited the potential role of CD47 blockade in producing anti-tumor effects [82–84]. Notably, macrophage removal restored tumor development after CD47 blockage, illustrating that macrophages play an indispensable role in preventing cancer cell growth following CD47 dampening [22]. There are four main mechanisms of CD47 blockade in targeting cancer cells (Fig. 3) [85, 86]. (1) Direct cancer cell killing: CD47 mAbs trigger tumor cell apoptosis via a mechanism independent of caspases [87]. (2) Macrophage-modulated antibody-dependent cellular phagocytosis (ADCP): Dampening of the CD47/SIRPα cross talk using CD47 mAb results in tumor cells phagocytic uptake by macrophages [88]. *Gloria H.Y. Lin* et al. reported that disrupting CD47/SIRPα cross talk triggers phagocytosis of tumor cells by all macrophage subsets, especially M1 and M2c macrophages [89]. Furthermore, *Gloria H.Y. Lin* et al. found that disrupting CD47/SIRPα cross talk triggers phagocytosis of tumor cells by a diverse panel of polarized macrophages and this process is required for the expression of FcγRs [89]. This implies that dampening CD47 effectively triggers the destruction of cancer cells by the heterogeneous population of macrophages observed in vivo. Primarily, increased cancer cell phagocytosis resulting from disruption of the CD47/SIRPα cross talk leads to enhanced presentation of antigens and CD8^+^ T cell proliferation in vitro. (3) DC-mediated presentation of antigens and T cell-modulated immune responses: CD47 mAbs promote tumor cell phagocytic ingestion by DCs coupled with subsequent presentation of antigens to CD8^+^ T cells, eliciting an anticancer adaptive immune response [90]. (4) NK cell-modulated antibody-dependent cellular cytotoxicity (ADCC) and complement-dependent cytotoxicity (CDC): SIRPα is a remarkable inhibitor of NK cell-modulated cytotoxicity, and CD47 mAbs destroy tumor cells by NK cell-modulated ADCC and CDC [91]. Therefore, blocking the CD47/SIRPα cross talk activates the innate and adaptive immune systems, leading to tumor cell destruction. Since CD47 mAbs activate the phagocytosis of macrophages, CD47 mAb therapy should be avoided in patients with hematological malignancy-associated hemophagocytic lymphohistiocytosis and monocyte/macrophage-related malignancies.

(3). **Clinical efficacy and adverse effects (AEs) of inhibiting the CD47/SIRPα axis in hematological malignancies**

Since 2015, several CD47/SIRPα mAbs or fusion proteins have been developed by different companies worldwide [33, 92, 93]. Their safety and efficacy in hematological malignancies are being demonstrated in clinical trials.

#### Myelodysplastic syndrome (MDS) and acute myeloid leukemia (AML)

In a phase I multicenter study (NCT02641002), the safety, tolerability, and clinical efficacy of CC-90002 as a monotherapy in AML and MDS were evaluated [94]. This research included 24 individuals with relapsed/refractory AML (R/R-AML) and four patients with high-risk R/R MDS. The patients received CC-90002 at 4 mg/kg (*n* = 6), 2 mg/kg (*n* = 4), 1 mg/kg *(n* = 6), 0.3 mg/kg (*n* = 6), and 0.1 mg/kg (*n* = 6). Four cases of dose-limiting toxicity (DLT) were reported in this study. Of these cases, one patient, receiving the 0.1 mg/kg dose, experienced disseminated intravascular coagulation and cerebral hemorrhage, both of grade 4; one patient, receiving the 0.3 mg/kg dose, had purpura of grade 3; one patient, receiving the 1 mg/kg dose, had congestive cardiac failure and acute respiratory failure, both of grade 4; and one patient, receiving the 4 mg/kg dose, had sepsis of grade 4. The most frequent (≥ 30%) any grade treatment-emergent adverse events (TEAEs) consisted of increased cough, alanine, anemia, and aminotransferase (32% each); increased aspartate aminotransferase and febrile neutropenia (36% each); thrombocytopenia (39%); and diarrhea (46%). A total of 23 patients (82%) had serious TEAEs, with general deterioration in physical health (*n* = 3), pneumonia (*n* = 4), bacteremia (*n* = 4), and febrile neutropenia (*n* = 10). TEAEs led seven participants (25%) to withdraw from the research. Overall, 82% required RBC transfusions, and CC-90002 therapy did not affect the patients’ ability to continue receiving RBC transfusions. The study’s findings suggested that CC-9002 monotherapy did not show good clinical efficacy in treating hematological malignancies. Furthermore, CC-9002 increased DLT. Consequently, research on CC-90002-AML-001 as a monotherapy was discontinued [95].

Nevertheless, studies have shown that magrolimab can be safely administered at a low “priming” dose. A transient anemia accompanied by compensatory reticulocytosis was observed in these studies, but severe anemia was not observed at subsequent higher maintenance doses [96, 97]. In addition, the updated ASCO abstract in 2022 also confirmed that HR-MDS patients treated with magrolimab in combination with AZA exhibit a tolerable anemia through priming and maintenance doses [98]. A phase IB study explored the clinical efficacy of magrolimab (Hu5F9) in combination with azacytidine (AZA) in MDS and AML patients [99]. Forty-three patients (18 MDS and 25 AML with a median age of 73 years) were given magrolimab combined with AZA [99]. Twenty-eight percent of patients harbored a TP53 mutation. The objective response rate (ORR) was 100% among the 13 evaluable MDS patients, while the ORR was 69% among the 16 evaluable AML patients [99]. TEAEs for magrolimab combined with AZA affected > 15% of patients: thrombocytopenia (26%), neutropenia (26%), and anemia (37%). Only one patient (2%) developed febrile neutropenia due to the treatment. Only one subject withdrew from the research study, owing to TEAE.

In a similar study conducted by the American Society of Clinical Oncology (ASCO) in 2020, 68 patients (39 MDS, 29 AML) with a median age of 72 years were treated using magrolimab combined with AZA. Twenty-seven percent had a TP53 mutation. The ORR was 91% among the 33 evaluable MDS patients, while the ORR was 64% among the 25 evaluable AML patients [100]. However, of the 12 AML patients with the *TP53* mutation, 75% had a CR + CRi, suggesting unique therapeutic effects in these patients [100]. Common TEAEs were infusion reaction (16%), thrombocytopenia (18%), anemia (38%), neutropenia (19%), and fatigue (21%). Treatment-linked febrile neutropenia was 1.5%. Only one case (1.5%) withdrew from treatment due to an TEAE. Patients who required RBC transfusions became transfusion-independent in 58% of MDS cases and 64% of AML cases. In conclusion, magrolimab combined with AZA is well tolerated and has durable efficacy for MDS and AML, particularly regarding the *TP53* mutation group, which is a poor prognostic group [100].

In a phase IB clinical trial reported by ASCO in 2022, magrolimab combined with AZA was evaluated for safety and efficacy in 72 AML patients with TP53 mutation [101]. All the patients are not suitable for intensive chemotherapy. The age ranges from 31 to 89 years, and the median age is 73 years [101]. The ORR was 48.6% among the 72 evaluable patients, including 33.3% CR, 8.3% CRi/CRh, 1.4% morphologic leukemia-free state (MLFS), and 5.6% partial responses (PR). It was found that the median durations of CR and CR/CRi were 7.7 months (95% CI: 4.7, 10.9) and 8.7 months (95% CI: 5.3, 10.9), respectively. In the 72 patients, the mOS was 10.8 months (95% CI: 6.8, 12.8) with an 8.3-month median follow-up [101]. The TEAEs were similar to those seen in other clinical trials of magrolimab combined with AZA. And a phase III clinical trial comparing this combination with standard of care in patients with TP53-mutant AML is currently ongoing (ENHANCE-2; NCT04778397).

Another phase IB clinical study from ASCO in 2022 reported the final results in 95 patients with untreated HR-MDS (NCT03248479) [102]. The age ranges from 28 to 91 years, and the median age is 69 years [102]. The ORR (33% CR) was 75% in all patients. Furthermore, the ORR was 79% (31% CR) in 61 MDS patients without TP53 mutation. And the ORR (40% CR) was 68% in 25 MDS patients with TP53 mutation [102]. The mPFS of all patients, TP53-wild-type patients, and TP53-mutant patients were 11.6 months, 11.8 months, and 11.0 months, respectively. The mOS of TP53-mutant patients was 16.3 months, while the mOS did not reach for all patients and TP53-wild-type patients. Additionally, the TEAEs were manageable in the present study [102]. In September 2020, the phase III ENHANCE trial (NCT04313881) began. Its purpose is to compare the efficacy and safety of AZA combined with magrolimab to AZA combined with a placebo (PBO) in previously untreated patients with HR-MDS [41]. Approximately 520 patients globally have been enrolled. It is the first phase 3 clinical trial of CD47 mAb in HR-MDS, and researchers await the results. Furthermore, the clinical trials of many distinct types of CD47 mAbs or SIRPα fusion proteins for the treatment of MDS and AML are also reported in our previous review [30]. In conclusion, CD47 mAbs or SIRPα fusion combined with other anti-tumor drugs could be an effective treatment option for AML or MDS patients.

#### Non-Hodgkin lymphoma (NHL)

A previous investigation assessed the clinical efficacy of magrolimab in conjunction with rituximab in R/R-NHL [42]. Twenty-two subjects were enrolled in the study, 15 with diffuse large B cell lymphoma (DLBCL), and seven with follicular lymphoma (FL). Patients were treated with a median of four prior treatments (range: 2 to 10), and 95% of them had a condition that was resistant to rituximab. Patients with NHL had a 50% ORR, those with DLBCL had a 40% ORR, and those with FL had a 71% ORR [42]. Anemia and infusion-linked responses were the most prevalent AEs. However, the 5F9 prime and maintenance dose approach helped reduce anemia. Dose-limiting side effects were uncommon [42].

ALX148 (NCT03013218) is a fusion protein composed of a CD47 blocker with an inactive human immunoglobulin Fc region [103]. This fusion protein was studied to treat CD20-positive B-cell-R/R-NHL patients in a phase I clinical study [103]. In this study, twenty patients with R/R-NHL were treated using ALX148 in conjunction with rituximab. The patients include 11 DLBCL cases, four mantle cell lymphoma (MCL) cases, three FL cases, and two marginal zone lymphoma (MZL) cases [103]. The maximum administered dose in this study was 10 mg/kg QW. No dose toxicities of ALX148 were seen, and the maximum tolerated dose (MTD) was not reached [103]. The total AE rate at any grade was 80% (16/20). The most frequent AEs consisted of rash (20%), anemia (10%), fatigue (10%), nausea (10%), neutropenia (10%), and decreased platelets (10%). However, only two patients experience grade 3 or 4 neutropenia [103]. All tumor histologies had a 35% ORR, with indolent (FL + MZL) histologies having a 40% ORR and aggressive (DLBCL + MCL) histologies having a 31% ORR. In patients with R/R-NHL, ALX148 combined with rituximab showed good tolerance and good objective responses [103].

TTI-621 binds minimally to human RBCs, thus alleviating the anemia associated with anti-CD47 mAbs [104]. A phase I clinical study (NCT02663518) of TTI-621 (SIRPα-IgG1 Fc) assessed the safety and activity of single-agent TTI-621 in R/R hematological cancers [105]. This study enrolled 164 patients, with 18 patients in the escalation group and 146 in the expansion group (combination of rituximab *n* = 35, combination of nivolumab *n* = 4, and monotherapy *n* = 107). The ORR was 13% for all patients. DLBCL had a 29% (2/7) ORR, T-NHL had a 25% (8/32) ORR with TTI-621 monotherapy, and DLBCL had a 21% (5/24) ORR with the combination TTI-621 and rituximab treatment [105]. Infusion-linked responses, thrombocytopenia, chills, and fatigue were among the AEs. It was easy to reverse thrombocytopenia (20%, grade 3) between doses, and it was not associated with bleeding. This clinical study enrolled a relatively large number of patients to better evaluate the safety and clinical efficacy of single-agent macrophage-targeted therapy in hematological malignancies. TTI-621 was well tolerated and exerted an anti-tumor effect as a monotherapy in patients with R/R B-NHL and T-NHL. The updates of the phase I study (NCT02663518) indicate that there were similar TEAEs with low-dose TTI-621 monotherapy (0.2 mg/kg to 0.5 mg/kg) in 214 patients with hematological malignancies [106]. Significantly, the ORR was 20% in 71 patients with NHL, with a 19.05% (8/42) occurrence of ORR with cutaneous T-cell lymphoma (CTCL), 18.18% (4/22) with peripheral T-cell lymphoma (PTCL), and 28.57% (2/7) with DLBCL. Furthermore, 24 patients with CTCL (18 patients with mycosis fungoides and six patients with Sézary syndrome) were enrolled into the high-dose group, including nine patients who received 2.0 mg/kg. Fifty percent of the patients had infusion-related reactions (13% Grade ≥ 3) and 33% (25% Grade ≥ 3) had thrombocytopenia, but all the TEAEs were manageable. The ORR was 20% (4/20) in the 20 patients who were evaluated, with 21.43% (3/14) of those with mycosis fungoides and 16.67% (1/6) of those with Sézary syndrome developing ORR [106]. Another multicenter phase 1 study reported on the safety and efficacy of TTI-621 in 35 patients (13 patients in the escalation group and 22 patients in the expansion group) with percutaneously accessible R/R mycosis fungoides or Sézary syndrome (NCT02890368) [107]. In this study, the dosage administered was up to 10 mg/kg; however, no dose-limiting TAEAs occurred. Significantly, 90% (26/29) of the evaluated patients had decreased Composite Assessment of Index Lesion Severity (CAILS) scores [106].

#### Hodgkin lymphoma (HL)

HL patients with high CD47 expression had a significantly lower event-free survival and OS compared to patients with low CD47 expression [108]. A clinical study of TTI-621 enrolled 24 patients with HL [105]. The TEAEs are similar to those experienced in other types of hematological malignancies. The ORR was 12.5% (3/24), and the disease control rate (DCR) was 62.5% (15/24). Furthermore, the ORR was 50% (2/4) in four evaluated HL patients who received TTI-621 in combination with nivolumab. Another study enrolled 14 patients with R/R lymphoma to evaluate the safety, tolerability, pharmacokinetics (PK), pharmacodynamics (PD), and clinical responses of IMM01 (SIRPα-IgG 1 fusion protein) [109]. There were no DLTs when the dose of IMM01 increased up to 1.0 mg/kg. In the present study, the most common TEAEs of IMM01 were thrombocytopenia (54%), neutropenia (36%), pyrexia (36%), and anemia (27%) [109]. One HL patient who had failed anti-PD-1 blockade treatment achieved PR, and one HL patient who had failed anti-PD-1 blockade treatment achieved a shrunk stable disease (SD) [109]. Furthermore, the half-life of IMM01 ranges between 53.8 h and 73.3 h, which suggests that a single dose can be administered for a longer period of time [109]. The sample size of the investigation into blocking CD47/SIRPα in HL treatment is still small and should be increased in future trials to observe the role of blocking CD47/SIRPα alone or in combination with other drugs in HL.

#### Multiple myeloma (MM)

Preclinical studies have demonstrated that blocking the CD47/SIRPα signaling pathway significantly enhances the killing effect of macrophages on MM in vitro and in vivo [110–112]. While clinical trials for the role of CD47 mAb in MM are relatively rare, none of eight patients with MM enrolled in a phase I clinical study (NCT02663518) of TTI-621 achieved ORR. TTI-622 (SIRPα-IgG4 Fc fusion protein, NCT03530683) and AO-176 (a humanized IgG2 anti-CD47 mAb, NCT04445701) are being evaluated as a monotherapy or a combination therapy with proteasome inhibitors and dexamethasone in R/R MM patients [113, 114]. Furthermore, *Edward Stadtmauer* et al. conducted a phase IB dose escalation and expansion study of CD47 mAb (TJ011133) for the treatment of R/R multiple myeloma on January 17, 2022 [115]. This clinical trial used CD47 mAb with or without dexamethasone plus an anti-myeloma regimen for the treatment of R/R MM [115]. The enrolled patients were divided into four groups for dosing, which included TJ011133, TJ011133 plus Pomalidomide and Dexamethasone; TJ011133 plus Carfilzomib and Dexamethasone; and TJ011133 plus Daratumumab and Dexamethasone. The primary outcome of this study is that there were DLTs with TJ011133, with or without dexamethasone and in combination with anti-myeloma regimens, in the participants with R/R-MM. The secondary outcomes include the percentages of the participants who achieved the best overall responses of the documented PRs or, even better, PFS, duration of response (DOR), and time to progression (TTP). This study is ongoing (NCT04895410), as more time is needed to observe the clinical and associated adverse effects of TJ011133 on MM.

In conclusion, CD47-targeted mAbs combined with current therapies may be an effective treatment option for hematological malignancies. Furthermore, various studies that are evaluating the clinical efficacies and TEAEs of CD47-targeted mAbs are in progress around the world. Nevertheless, *Gilead Sciences, Inc.* paused enrollment in some trials of magrolimab for the treatment of AML and MDS, following concerns about safety issues among those receiving it in combination with AZA [116]. In addition, CD47 products with improved safety in the future are expected to be more effective in treating hematological malignancies. The selected ongoing CD47/SIRPα programs for hematological malignancies are presented in Table 1.

**Table 1:** Clinical trials of CD47/SIRPα-targeted agents in hematological malignancies

	Type of mAb	Subclass of IgG	Initial time of clinical studies	Phase	Type of tumors	Treatment programs	ClinicalTrials.gov Identifier
Hu5F9-G4	mAb	IgG4	2014.8	Phase II	MDS and AML	Combined therapy	NCT02216409
Hu5F9-G4	mAb	IgG4	2020.9	Phase III	Higher-risk MDS	Combined therapy	NCT04313881
Hu5F9-G4	mAb	IgG4	2021.11	Phase II	R/R cHL	Combined therapy	NCT04788043
Hu5F9-G4	mAb	IgG4	2021.12	Phase I	R/R B-Malignancies	Combined therapy	NCT04599634
Hu5F9-G4	mAb	IgG4	2022.05	Phage III	TP53 Mutant AML	Combined therapy	NCT04778397
Hu5F9-G4	mAb	IgG4	2022.9	Phase IB/II	MDS and AML	Combined therapy	NCT05367401
TTI-621	SIRPα fusion protein	IgG1	2016.1	Phase IA/IB	Hematological Malignancies	Monotherapy	NCT02663518
TTI-621	SIRPα fusion protein	IgG1	2021.10	Phase IB	MM	Combined therapy	NCT05139225
TTI-622	SIRPα fusion protein	IgG4	2018.5	Phase IA/IB	Hematological Malignancies	Combined therapy	NCT03530683
TTI-622	SIRPα fusion protein	IgG4	2021.1	Phase IB	R/R MM	Combined therapy	NCT05139225
ALX148	IRPα fusion protein	IgG1	2017.2	Phase I	Lymphoma	Combined therapy	NCT03013218
ALX148	SIRPα fusion protein	IgG1	2020.1	Phase I/II	MDS	Combined therapy	NCT04417517
ALX148	SIRPα fusion protein	IgG1	2021.1	Phase I/II	B-NHL	Combined therapy	NCT05025800
ALX148	SIRPα fusion protein	IgG1	2021.5	Phase I/II	AML	Combined therapy	NCT04755244
AK117	mAb	IgG4	2020.4	Phase I	Lymphoma	Monotherapy	NCT04349969
AK117	mAb	IgG4	2021.1	Phase I	Lymphoma	Monotherapy	NCT04728334
AK117	mAb	IgG4	2021.5	Phase I/II	Higher-risk MDS	Combined therapy	NCT04900350
AK117	mAb	IgG4	2021.7	Phase IB/II	AML	Combined therapy	NCT04980885
Gentulizumab	mAb	Un	2021.4	Phase I	NHL	Combined therapy	NCT05221385
Gentulizumab	mAb	Un	2021.4	Phase I	R/R-AML or MDS	Monotherapy	NCT05263271
IMM-01	SIRPα fusion protein	IgG1	2019.9	Phase I	Lymphoma	Monotherapy	ChiCTR1900024904
IMM-01	SIRPα fusion protein	IgG1	2022.1	Phase I/II	MDS and AML	Combined therapy	NCT05140811
SRF231	mAb	IgG4	2018.3	Phase IA/IB	lymphoma/CLL	Combined therapy	NCT03512340
SHR1603	mAb	IgG4	2018.1	Phase I	Lymphoma	Combined therapy	NCT03722186
IBI188	mAb	IgG4	2018.12	Phase I	lymphoma	Combined therapy	NCT03717103
IBI188	mAb	IgG4	2020.8	Phase IB	Newly Diagnosed HR-MDS	Combined therapy	NCT04511975
IBI188	mAb	IgG4	2020.9	Phase IB	AML	Combined therapy	NCT04485052
IBI188	mAb	IgG4	2020.9	Phase IB	Newly Diagnosed HR-MDS	Combined therapy	NCT04485065
TJC4	mAb	IgG4	2019.5	Phase I	lymphoma	Combined therapy	NCT03934814
TJC4	mAb	IgG4	2021.6	Phase IB	MDS and AML	Combined therapy	NCT04912063
TJC4	mAb	IgG4	2022.1	Phase IB	Multiple Myeloma	Combined therapy	NCT04895410
ZL-1201	mAb	IgG4	2020.5	Phase I	Lymphoma	Combined therapy	NCT04257617
IMC-002	mAb	IgG4	2020.6	Phase I	Lymphoma	Combined therapy	NCT04306224
AO-176	mAb	IgG2	2020.11	Phase I/II	Multiple Myeloma	Combined therapy	NCT04445701
CC-95251	mAb	IgG4	2019.2	Phase I	Hematological Cancers	Combined therapy	NCT03783403
CC-95251	mAb	IgG4	2022.1	Phase I	MDS and AML	Combined therapy	NCT05168202
CD47	mAb	Un	2021.12	Single-arm	Recurrent AML After Transplantation	Combined therapy	NCT05266274

*Un* Unknown

(4). **Challenges and strategies for applications of CD47 mAbs**

Given the anti-tumor mechanisms and AEs of CD47/SIRPα blockades found in hematological malignancies’ treatment research [30], several points should be considered during drug design and development: 1) Can CD47 antibodies interact with RBCs? RBCs also express CD47; therefore, the antibodies may interact with different RBCs, leading to agglutination and lysis of RBCs. 2) Are the antibodies IgG1? Fc units of IgG1 can interact with FcγR expressed by macrophages, leading to phagocytosis of RBCs by macrophages. 3) A subpopulation of T cells also expresses CD47. Can the antibodies interact with CD47 expressed by T cells (which can also lead to T cell apoptosis)? 4) If CD47 mAb is the IgG4 subtype, which does not have an Fc effect, it cannot fully activate phagocytosis by macrophages. The clinical effects dramatically decrease, and no satisfactory clinical results can be achieved with monotherapy of these antibodies. There is a need to identify appropriate subclasses of IgG and optimize single-chain variable regions to develop the best therapeutic antibodies [117, 118].

In light of these issues, the following points should be noted: 1) The selected antibody cannot interact with RBCs (e.g., TTI-621, TTI-622, TJC4, IMM01, and AK117). 2) The selected antibody cannot induce T cell apoptosis. (3) The selected antibody should possess a CD47- or SIRPα-inhibiting target and activating Fc effect, which is capable of enhancing anti-tumor activity [119] (e.g., TTI-621 and IMM01). 4) Additionally, macrophages express “eat me” signals or surface markers such as calreticulin, signaling lymphocytic activation molecule family member 7 (SLAMF7), Mertk, and Axl [72, 120, 121]. Knockout of SLAMF7 in mice remarkably inhibits macrophage-mediated phagocytosis potentiated by CD47 blockade in many B cell- and myeloid cell-derived cancer cell lines [121]. The discovery of the activation receptor has significant implications for CD47’s clinical application. Thus, the activation of the receptors can be applied as a biomarker to estimate the clinical responses of a phagocytic checkpoint blockade. Targeting phagocytosis to activate the receptor is a reasonable approach for promoting tumor clearance by macrophages in combination with phagocytosis checkpoint blockade.

(5). **Other potential therapeutic strategies based on CD47/SIRPα blockades**

#### Combination of CAR-T cells and CD47/SIRPα blockers

In the treatment of hematological malignancies, CAR-T cells have shown outstanding results. However, short disease-free survival times and high recurrence rates are significant obstacles [122]. The main mechanisms of current CAR-T cell therapy failure include loss of target antigens, impaired T cell function, and a complex immunosuppressive microenvironment [122]. Given the potential antigen-presenting advantages of CD47 blockade in improving adaptive immunity function when combined with CD8^+^ T cells [90], further research into the combination of CD47 blockade with CAR-T cells for cancer therapy is critical. *Huanpeng Chen* et al. designed a CAR-T cell-secreting CD47 blocker SIRP-Fc fusion protein (dubbed Sirf CAR-T) to enhance the therapeutic efficacy of CAR-T cells in solid tumor treatment [123]. In numerous syngeneic immunocompetent tumor models, *Chen* et al. discovered that Sirf CAR-T cells drastically reduced tumor burden and improved the survival time of tumor-bearing mice [123]. They found that Sirf CAR-T cells induced more central memory T cells, improved CAR-T cell persistence in tumor tissue, and reduced PD-1 expression on the CAR-T cell surface to identify the mechanisms of Sirf CAR-T cells against malignancies [123]. They also found that Sirf CAR-T cells might change the TME by reducing myeloid-derived stem cells (MDSCs) and boosting CD11c^+^ DCs and M1-polarized macrophages in the tumor [123]. In conclusion, *Chen* et al.’s research reveals that the SIRP-Fc improves the anti-tumor efficacy of CAR-T cells and suggests a blocking CD47/SIRPα signaling influence on the role of CAR-T cells. These outcomes could rationalize using CD47 blockades in combination with CAR-T cells [110], providing a novel approach for effective tumor immunotherapy. Prior to this study, no clinical studies evaluated CD47 blocker combined with CAR-T cell therapy in cancers. Further studies should evaluate the influences of CD47 inhibitors in combination with CAR-T cell therapy in hematological malignancies.

#### CD47/SIRPα-targeted antibody–drug conjugate (ADC)

Antibody–drug conjugates (ADCs) are formed by a mAb with a payload through a linker. It has both the powerful killing effect of the payload and the tumor targeted of antibody drugs [124, 125]. Since the first ADC (gemtuzumab ozogamicin) was approved for the treatment of CD33-positive AML, nine distinct types of ADC drugs were approved as cancer treatments by the FDA [126]. Recently, CD47/SIRPα-targeted ADCs have also gradually been deployed. While, only the CD47-targeted ADC, SGN-CD47M has entered the clinical stage, and phase I clinical trials for the treatment of advanced solid tumors are underway (NCT03957096) [127]. In addition, TAC-002 and BYON4228 are both SIRPα antibody-conjugated TLR9 agonists, which are in preclinical development. More studies are also required to evaluate the effect of CD47/SIRPα-targeted ADCs on hematological malignancies.

### PD-1/PD-L1 axis

*Sydney R. Gordon* et al.’s 2017 study documented a new role of the PD-1/PD-L1 signal pathway in modulating the phagocytic capacity of TAMs [43]. This study suggests that the PD-1/PD-L1 signaling pathway in macrophages could also function as the “don’t eat me” signal. They exhibited that PD-1 was expressed in mouse and human TAMs. Furthermore, PD-1 expression levels increased through cancer progression in mice models and high TNM staging in primary human malignancies [43]. PD-1^+^ TAMs exhibit an M2-like macrophage phenotype and display a considerably reduced phagocytic potency against cancer cells. However, inhibition of the cross talk between PD-1 and PD-L1, either via PD-1 or PD-L1 blockade, results in an anticancer immune response in mice that lack T cells, B cells, and NK cells but have functional macrophages [43]. The phagocytic function of PD-1^+^ TAMs is rescued in *PD-L1*^−/−^ mice in vivo. However, there is no significant difference in phagocytosis of PD-1^−^ TAMs between PD-L1-expressing and knockout tumor cells [43]. These findings strongly suggest that PD-L1-expressing cancer cells can evade TAM-mediated phagocytosis. Previous investigations have shown that PD-1 expression inhibits a wide variety of immune cells in the TME, consisting of T cells [128], B cells [129], NK cells [130], and DCs [131]. *Sydney R Gordon* et al. extended their study to include macrophages, further emphasizing the essential function of PD-1 expression in immune system balance across the innate and adaptive immune systems [43].

Anti-PD-1/PD-L1 treatments have been explored in various hematological cancers, but there is still a need to establish why blocking the PD-1/PD-L1 axis demonstrates clinical efficacy only in a minority of cancer types [1, 5, 132]. The influences of PD-1 blockade on TAMs in human cancer should not be ignored as it may aid the search for new disease biomarkers or therapeutic combinations. For example, PD-1 blockade exhibited high clinical responses in HL patients despite the heterogeneous expression of PD-1 on tumor-infiltrating lymphocytes (TILs). Frequently, PD-1 blockade compromised class I major histocompatibility complex (MHC-I) surface expression on tumor cells, which potentially precludes the T cell-mediated anti-tumor immune response mechanism [132]. This inconsistency in the PD-1/PD-L1 blockade could partially be explained by the TAMs-mediated anti-tumor immune effect on HL [132]. These findings indicate that adaptive and innate immune checkpoints need further investigation. The findings also suggest that the study of innate and adaptive immunity can provide a novel understanding of the heterogeneity of clinical responses to immune checkpoint inhibitors.

### MHC-I/LILRB1/2 axis

MHC-I expressed by tumor cells also inhibits macrophage-mediated phagocytosis [44]. MHC-I comprises HLA α-chains and β_2_-microglobulin (β_2_-M) chains [133]. Some tumor cells highly express the MHC-I complex to inhibit macrophage-mediated phagocytosis [44]. To better understand the process of MHC-I-induced suppression of phagocytosis, researchers have identified the receptors utilized in detecting MHC-I. The study has reported that two leukocyte immunoglobulin-like receptor (LILR) family members, LILRB1 and LILRB2, couple with MHC-I and harbor immunoreceptor tyrosine-based inhibitory motifs (ITIMs), aiding in the inhibition of intracellular signal transduction [44]. LILRB1 and LILRB2 are considered candidates for the MHC-I-modulated inhibition of phagocytosis. An analysis of human monocytes showed that LILRB1 was expressed in most monocytes, while only a small subpopulation of LILRB2^+^ was detected [44]. TAMs in several tumors highly express LILRB1 but not LILRB2. Treatment with LILRB1 mAb has been shown to significantly increase the anti-CD47-triggered phagocytosis of MHC-I^+^ cells but not MHC-I^−^ cells [44]. In contrast, anti-LILRB2 mAb have no considerable impact on phagocytosis of either MHC-I^+^ cells or MHC-I^−^ cells [44]. The MHC-I/LILRB1 signaling axis is a “don’t eat me” signaling pathway, and inhibition of either LILRB1 or MHC-I may greatly enhance tumor cell phagocytosis. Although MHC-I or LILRB1 blockade promotes macrophage-mediated phagocytosis, it does not extensively inhibit tumor growth in immunocompetent mice, indicating some of the limitations of this therapy.

Previous studies have shown that blocking LILRB2 using therapeutic antibodies can boost macrophages’ maturation and strengthen their pro-inflammatory phenotype [46]. Additionally, LILRB2 blockade combined with anti-PD-L1 mAb enhances the phagocytosis of TAMs and increases their anti-tumor effects in transgenic mice, which can express human LILRB2 on myeloid cells [46]. However, it remains unclear whether blocking LILRB2 promotes direct or indirect phagocytosis of TAMs. Although LILRB1 and LILRB2 dock to MHC-I, it is unknown whether LILRB2 ligand cross talk also works as a phagocytosis checkpoint [134, 135]. Anti-LILRB2 mAbs have been examined in a phase I clinical trial of malignancies (JTX-8064, INNATE) [136]. However, further investigations are needed to examine MHC-I expression patterns in hematological malignancies and LILRB1/2 expression patterns in associated TAMs. Based on phase I clinical data, this phagocytosis regulator is a promising target for enhancing ADCP against hematological malignancies [1]. Clinical research studies are also needed to examine the clinical efficacy of blocking the MHC-I/LILRB1/2 axis in hematological malignancies. Finally, LILRB1 is also expressed on several other distinct types of human immune cells, including B cells, DCs, NK cells and T cells [137]. Significantly, *Chen* et al. found that LILRB1 mAb enhanced anti-tumor activity of NK cells in multiple myeloma, leukemia and lymphoma [137]. Therefore, the study of LILRB1-mediated innate and adaptive immunity should be considered in the future.

### CD24/sialic acid-binding Ig-like lectin 10 (SIGLEC-10) axis

CD24 is also called the heat-stable antigen or small-cell lung cancer cluster-4 antigen. It belongs to a glycosylphosphatidylinositol-anchored surface protein with a high glycosylation level [138, 139]. The expression level of CD24 is upregulated across almost all tumor types but not normal tissues [140–144]. Furthermore, in tumor tissues, the expression of CD24, another known innate immune checkpoint, is notably strong compared to the expression of CD47 [47, 140]. The highest upregulation of CD24 expression has been reported in triple-negative breast cancer (TNBC) and ovarian cancer patients, who showed decreased recurrence-free survival (RFS) time and OS, respectively [47, 140]. Therefore, CD24 constitutes a “don’t eat me” signal and has been defined as an immune checkpoint via cross talk with the inhibitory receptor SIGLEC-10 on macrophages in the TME [47, 140]. These mechanisms have also been confirmed by single-cell RNA-seq results and fluorescence-activated cell-sorting assays using primary ovarian and breast cancer tissues [47, 140]. CD24 is especially upregulated in tumor cells, while SIGLEC-10 expression occurs in a subpopulation of macrophages in the TME, suggesting a potential cross talk between CD24 and SIGLEC-10 [47, 140].

*Amira A Barkal* et al. investigated the role of the CD24/SIGLEC-10 axis in modulating macrophage-triggered phagocytosis [47]. Studies have shown that macrophages in a co-culture system more readily phagocytose CD24^−/−^ MCF-7 cells than wild-type MCF-7 cells in vitro [47]. Additionally, *SIGLEC-*10^−/−^ macrophages also show significantly enhanced phagocytosis of the wild-type MCF-7 cells, further confirming that the CD24/SIGLEC-10 axis exerts a “don’t eat me” effect [47]. Anti-CD24 antibody therapy is also reported to substantially increase phagocytosis of wild-type MCF-7 cells in macrophages [47]. The pro-phagocytic effects of inhibiting the CD24/SIGLEC-10 axis are higher than the CD47 blockade treatment [47]. Another significant experimental result indicates that *SIGLEC-*10^−/−^ macrophages inhibit the phagocytosis effects of the anti-CD24 antibody, suggesting that CD24 blockade functions by disrupting the cross talk between CD24 and SIGLEC-10 [47]. An in vivo xenograft model experiment indicated that CD24-knockout MCF-7 cells had a markedly lower tumor burden than the wild-type group [47]. Moreover, macrophage depletion considerably inhibited the reduced tumor burden in CD24-knockout mice but not in wild-type mice, indicating that the anti-tumor effect may be attributed to macrophage-mediated phagocytosis [47]. These findings suggest that the CD24/SIGLEC-10 axis is a phagocytosis-related innate immune checkpoint that modulates anti-tumor immunity by regulating macrophage-mediated phagocytosis, demonstrating that CD24 blockade is a potential immunotherapy approach.

Previous preclinical investigations have shown that blocking CD24 has abundant potential in anti-tumor applications. Given that AML has a low expression of CD24, and acute lymphoblastic leukemia (ALL) and DLBCL have a moderate/high expression of CD24, it stands to reason that ALL and DLBCL may effectively respond to anti-CD24 treatment [47]. Significantly, *Andrea Aroldi* et al. reported on a functional study that has shown an improvement of phagocytosis through CD24/SIGLEC-10 axis inhibition in MCL [145]. Several MCL cell lines (e.g., NALM-6, Jeko-1, Granta-519, and Mino) express surface CD24, and immunosuppressive-induced M2-like macrophages demonstrated increased SIGLEC-10 expression [145]. In addition, *Andrea Aroldi* et al. performed a phagocytic assay via M2-like macrophages co-cultured with MCL cell lines. CD24 mAb was found to increase macrophage phagocytosis [145], which suggests a potential immunotherapeutic target in MCL with the aim of improving innate immunity via disruption of the CD24/SIGLEC-10 axis. Another in vitro study found that CD24 mAb removed more than 90% of MCL cell lines [146]. Furthermore, CD24 mAb triggered phagocytosis in primary patient-derived MCL cells by autologous macrophages [146]. Treatment for in vitro MCL cell lines with CD24 mAb was superior to CD47 mAb, which suggests that CD24 mAb may be more effective in treating MCL than CD47 mAb [146]. Despite these studies, in vivo studies are also required to confirm these in vitro efficacies documented for MCL. However, no clinical investigations have exhibited the efficacy of anti-CD24 in hematological malignancies; therefore, further studies are needed.

**Fig. 4:**
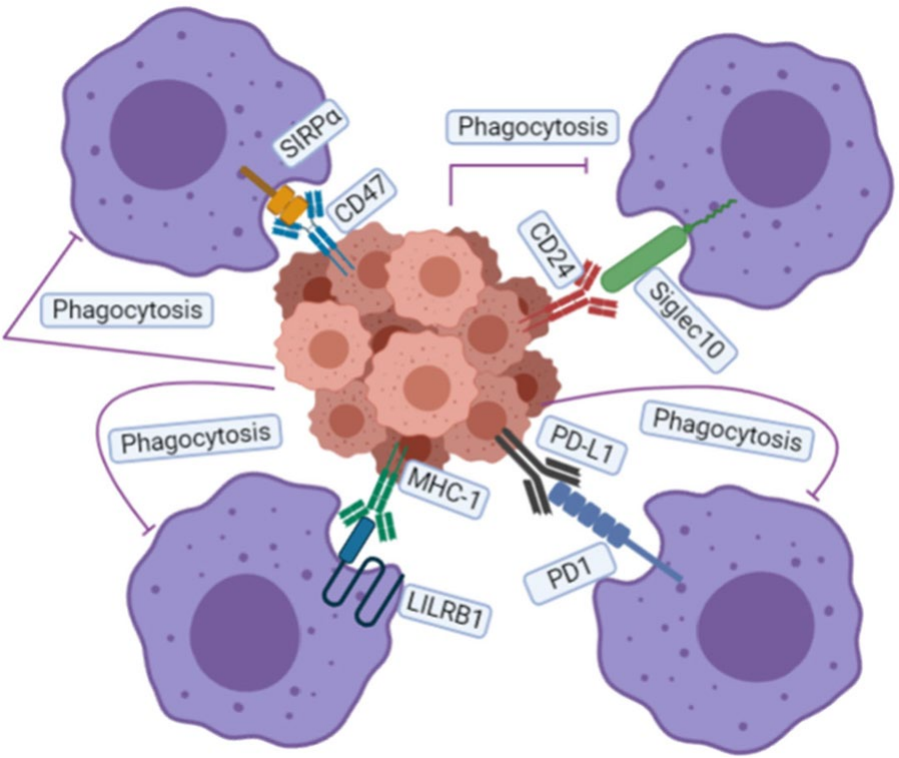
Main “Don’t Eat Me” signals discovered so far: The CD47/SIRPα axis, PD-1/PD-L1 axis, MHC-I/LILRB1 axis, and CD24/SIGLEC-10 axis all play significant roles in inhibiting cancer cell engulfment, and disruption of these interactions can enhance the phagocytosis of macrophages

In conclusion, there are four major phagocytosis checkpoint blockades (Fig. 4). In hematological malignancies, those that block the CD47/SIRPα axis and PD-1/PD-L1 axis have demonstrated promising clinical responses. However, larger clinical trials with longer follow-up times are required to confirm these findings. And the TEAEs of distinct CD47 products should also be considered in future. In addition, preclinical studies have shown potential therapeutic effects for another two phagocytosis checkpoints (MHC-I/LILRB1/2, CD24/SIGLEC10) in tumors. Still, more clinical studies are needed to examine the effects of these phagocytosis checkpoints on distinct types of hematological malignancies. Identifying heterogeneity in “don’t eat me” signal expression in different hematological malignancies indicates this area of research requires further investigation to select appropriate therapeutic strategies precisely. Because the anti-tumor influences of phagocytosis checkpoint blockades depend on the presence of macrophages [22], it is necessary to detect the number of macrophages infiltrated into the microenvironment of hematological malignancies and identify biomarkers. These steps can help identify patients who may benefit the most while avoiding substantial toxicities. Furthermore, preclinical studies have demonstrated that TTI-621 triggers phagocytosis of blood cancer cells through all macrophage subsets [104]. Therefore, macrophage-mediated phagocytosis checkpoint blockades in combination with therapeutic strategies to increase macrophage numbers may enhance the efficacy and utility of these therapies.

## Elimination of macrophages using either CSF1 or CSF1R inhibitors

As mentioned previously, TAMs constitute the most abundant immune cells in the TME and generally play a pro-tumoral role in clinical studies and experimental mouse models [147]. TAMs enhance tumor growth via numerous mechanisms [148]. Consequently, elevated numbers of TAMs are closely associated with the progression of many cancers [29]. On this basis, the deletion of macrophages is theoretically a potential treatment for hematological malignancies [3].

Studies have shown that *CSF1R* mRNA expression is restricted to myeloid cells [149]. More specifically, the proliferation, differentiation, and survival of macrophages are regulated by the *CSF1R* and its ligands, CSF1 and IL-34 [150, 151]. The deletion of *CSF1R* in mice or rat results in a deficiency of macrophage populations in most tissues [48, 152, 153]. Furthermore, tissue macrophages can be depleted via treatment using anti-CSF1R antibodies [154, 155] or CSF1R kinase inhibitors [156]. Unlike phagocytosis checkpoints, targeting the CSF1/CSF1R axis can generate a specific deletion of macrophages in the entire body, including TAMs in the TME.

Prompted by previous studies, clinical trials are underway in which small molecules and mAbs targeting CSF1R or its CSF1 ligand are being tested as a monotherapy and in combination with conventional therapies. There are three types of therapies targeting the CSF1/CSF1R axis: Pexidartinib (PLX3397), an oral tyrosine kinase CSF1R inhibitor; c-KIT, a mutant FMS-like tyrosine kinase-3 (FLT-3); and the platelet-derived growth factor receptor-β (PDGFR-β) [157, 158]. Additional compounds targeting the CSF1R include ARRY-382 [159], GW-2580 [160], PLX-3397 [161], BLZ-945 [148], JNJ-40346527 [162], and so on. Some mAbs in clinical development include emactuzumab [163], AMG-820 [164], and PD-0360324 [165]. Of these antibodies, PD-0360324 is the only compound targeting the CSF1 ligand.

A phase I/II study of 21 classic Hodgkin’s lymphoma (cHL) patients treated with JNJ-40346527 [162] demonstrated the best overall response, CR, occurred in only one patient, while SD occurred in 11 patients. The most common AEs included nausea, headache, and pyrexia. In summary, JNJ-40346527 was well tolerated by individuals with cHL, and early findings illustrate its modest effect as monotherapy for cHL.

Although macrophages are well known for their innate immune responses, there is increasing evidence of other roles macrophages play, including regulating the hematopoietic microenvironment and homeostasis, regulating erythropoiesis, mediating tissue repair, influencing metabolism, and overseeing the maturation of embryonic tissue [72, 166–168]. Accordingly, deletion of total macrophages by CSF1 or CSF1R inhibitors may result in numerous AEs; therefore, significant attention should be given to AEs that may arise during clinical trials. Since the anti-tumor effects of phagocytosis checkpoint blockades depend on the presence of macrophages, these blockades combined with the deletion of macrophages are not recommended for clinical application.

## CD47/SIRPα-targeted bispecific antibodies (BsAbs)

TAMs, a specific macrophage subpopulation, constitute the most abundant part of immune cells in almost all TME [169, 170]. However, TAM-targeted treatments have reported limited success in terms of clinical responses for the following reasons: lower drug aggregation in the tumor, insufficient suppression of signaling transduction, induction of feedback signaling transduction that results in resistance to monotherapy, and systemic dose-dependent toxicities [171]. In light of these obstacles, CD47/SIRPα-targeted BsAbs may be another promising strategy to fight cancer [172]. BsAbs can recognize and bind to two distinct antigens or epitopes simultaneously, providing specific and accurate targeting of tumor cells and reducing off-target toxicity [173]. IgG-based BsAbs have been generated based on this theory to block CD47 and specific tumor biomarkers or other immune cell biomarkers. These BsAbs include anti-CD47/CD20 [53], anti-CD47/CD19 [59], CD47/CD33 [60], CD47/PD-L1 [61], CD47/PD-1 [62], and so on.

### CD47/CD20 BsAb

CD47/CD20 BsAbs bind CD20 and CD47 monovalently. They have a lower affinity for CD47 than the parental antibody but maintain robust docking to CD20 [53, 54]. These properties enable BsAbs to attach specifically to CD47^+^CD20^+^ tumor cells, resulting in phagocytosis. *In vitro* studies reveal that CD47/CD20 BsAbs stimulate tumor cell phagocytosis in a CD20-dependent approach [53, 54]. Essentially, these in vivo studies have also indicated that the treatment of human NHL-inserted mice with CD47/CD20 BsAbs reduces lymphoma burden and prolongs the OS of mice [53, 54]. Therefore, BsAbs can produce synergistic treatment efficacy in therapies that combine anti-CD47 and anti-CD20 [53, 54].

In addition, Shanghai ImmuneOnco Biopharmaceuticals Co. (ImmuneOnco) developed a bispecific recombinant antibody trap named IMM0306. IMM0306 is a recombinant human SIRPα and anti-CD20 antibody fusion protein [174]. It can simultaneously target CD47 and CD20 on B cells but avoids docking to human RBCs in vitro. IMM0306 exhibits a strong pro-phagocytosis influence compared to CD47^+^CD20^+^ target cells and an even stronger ADCC influence than rituximab. Treating lymphoma-transplanted SCID mice with IMM0306 significantly hampered tumor growth and resulted in the complete remission of tumor cells in 62.5% of mice. This therapy was remarkably effective compared to rituximab alone or the co-administration of rituximab and SIRPα-Fc (IMM01) [174]. In conclusion, the research revealed that IMM0306 may be a promising approach for developing CD47-targeted immunotherapy. It allows for the targeted avoidance of RBC-modulated antigen drops and the onset of anemia while still exhibiting substantial anti-tumor efficacy. IMM0306 is now undergoing a phase I clinical investigation (NCT04746131). JMT601, designed by the China Shijiazhuang Pharmaceutical Company (CSPC), is another recombinant human SIRPα and anti-CD20 antibody fusion protein. A phase I study of JMT601 (NCT04853329) is ongoing. However, no clinical data have been published for CD47/CD20 BsAb.

### CD47/CD19 BsAb

TG-1801 (also known as NI-1701) is a fully humanized IgG1 BsAb designed to target and deplete B cells through various mechanisms [57]. In an in vitro study, TG-1801 bound to B cells in whole blood and specifically blocked CD47/SIRPα cross talk on CD19-expressing cells [57]. TG-1801 mediated the successful killing of primary and hematological cancer cell lines (Raji, Ramos, MEC-2, NALM-6, and SUDHL-4) through ADCP and ADCC [57]. Essentially, the in vitro anti-tumor activity extends in vivo killing efficacy*.* This study used a Raji B cell–transplanted NOD/SCID mouse model. TG-1801 led to the inhibition of tumor proliferation and a considerable increase in median OS compared to isotype control-treated mice [57]. The anti-tumor effects of TG-1801 were also demonstrated in a patient-derived xenograft model of B-ALL, where TG-1801 reduced tumors across the various organs tested [57]. The study suggests that TG-1801 offers an alternative treatment for patients who are resistant, refractory, or both to anti-CD20 therapy. A clinical investigation is currently being performed to validate the safety and efficacy of this BsAb (NCT04806035). Furthermore, *Emmanuel Normant* et al. explored potential synergies between TG-1801 and ublituximab alone, or thresholdisib alone, or ublituximab plus thresholdisib in B-NHL via in vitro and in vivo experiments [175]. Significantly, the tumor growth inhibition (TGI) for TG-1801 in combination with umbralisib alone, ublituximab alone, and ublituximab plus thresholdisib were 85%, 93%, and 93%, respectively [175]. Intriguingly, the anti-tumor effect of the different combinations of TG-1801 was associated with higher levels of mouse macrophage infiltration within tumors, as well as the upregulation of G-protein coupled receptor EBI2/GRP183. Additionally, the EBI2 small-molecule inhibitor NIBR189 inhibits the ADCP, B cell cytoskeleton remodeling, and inflammatory cytokine production induced by TG-1801 [175]. Taken together, these data suggest a combination strategy of TG-1801 with other anti-B-cell mechanisms, such as umbralisib and ublituximab, for the treatment of B-NHL [175]. Further clinical studies are also required to evaluate the combination strategy of TG-1801 with other anti-B-cell drugs.

### CD47/4-1BB BsAb

Targeting CD47 stimulates the innate immune system, in particular. Thus, therapy with CD47 mAbs enhances antigen presentation in the presence of MHC via macrophages, and DCs, thereby activating T cell cross-priming in mouse models [176]. Based on this rationale, a novel BsAb (DSP107) that inhibits the CD47 signaling pathway while simultaneously stimulating anti-tumor T cell immune response has been designed [177, 178]. DSP107 exhibited a high binding affinity for both human CD47 and 4-1BB [177]. DSP107 also blocked the cross talk of SIRPα with CD47 and triggered phagocytosis in several lymphomas, leukemia, and carcinoma cell lines in vitro [177]. By binding CD47 on cancer cells, DSP107 blocks the CD47/SIRPα cross talk, thereby inducing phagocytosis of cancer cells [178, 179]. Additionally, DSP107 binds to 4-1BB, a co-stimulatory receptor that is increased in response to T cell receptor (TCR)/MHC cross talk and a proven surrogate biomarker for the tumor-reactive T cell subset in the TME [178, 179]. Furthermore, DSP107 triggers 4-1BB signaling only after binding to CD47 [178, 179]. Taken together, DSP107 unleashes innate and adaptive immune responses to target the tumor site. In vitro, DSP107 alone or combined with rituximab triggers substantial phagocytosis of numerous DLBCL cancer cell lines and primary patient-derived DLBCL cells through macrophages [178, 179]. Additionally, 4-1BB activation is only observed following DSP107 binding to human CD47, and the induction of 4-1BB co-stimulatory signaling triggers prominent T cell proliferation to augment T cell cytotoxicity in vitro [178, 179]. Injecting PBMCs into mice with generated SUDHL6 xenografts accompanied by DSP107 treatment triggers a considerable decrement in tumor size compared to PBMC-only treatment [178, 179]. Thus, DSP107 induces innate and adaptive anti-tumor immunity, potentially making it useful in treating DLBCL, leukemia, and other diseases. Despite the lack of clinical results for DSP107 in hematological malignancies, the safety and efficacy of DSP107 in advanced solid tumors have been confirmed at the 2022 ASCO meeting [180]. A clinical trial is ongoing to assess DSP107’s safety and efficacy in the treatment of MDS, AML, and T cell lymphoproliferative disease (NCT04937166).

### CD47/CD33 BsAb

CD33 is over-expressed in 90% of patients with AML [181]. Commonly, CD47 is also over-expressed in patients with AML [33]. Therefore, *Jerome Boyd-Kirkup* et al. constructed a bispecific anti-CD47xCD33 antibody (HMBD004) [60]. HMBD004 exhibited preferential binding of CD47^+^CD33^+^ cells. HMBD004 significantly inhibits CD47/SIRPα cross talk and the induction of phagocytosis in vitro [60]. However, HMBD004 does not bind to RBCs in vitro, suggesting a lower incidence of hemagglutination [60]. Additionally, in vivo cell line-derived xenograft mouse models of AML were created by subcutaneously implanting NCr nude mice with the CD47^+^CD33^+^ AML cell line HL-60. The treatment of these animals with HMBD004 resulted in a reduction in tumor burden and an increase in PFS. In conclusion, these findings indicate that HMBD004 enhances the specificity, efficacy, and safety of CD47 mAb therapy in AML. Therefore, clinical studies on HMB004 are worthwhile.

### CD47/PD-L1 BsAb

Tumor cells express both PD-L1 and CD47, whereas most normal cells have limited or undetectable expression of PD-L1. The distinct co-expression pattern of PD-L1 and CD47 in cancers compared to normal tissues provides a rationale for designing BsAbs that can selectively recognize PD-L1^+^CD47^+^ tumor cells and block their CD47 signaling to trigger the engulfment of double-positive cancer cells. While the monovalent CD47 antibody arm binds CD47 and triggers strong tumor cell phagocytic activity, it has little effect on normal human CD47 single positive cells [61]. IBI322, a CD47/PD-L1 BsAb, was generated by blocking the PD-L1 signaling pathway with the bivalent single-domain PD-L1 antibody arm [61]. Numerous preclinical studies have examined the safety and efficacy of IBI322, both in vitro and in vivo [61, 182, 183]. IBI322 selectively binds CD47^+^PD-L1^+^ tumor cells, effectively inhibits CD47/SIRPα signaling, and instigates intense phagocytosis of CD47^+^PD-L1^+^ tumor cells by macrophages in vitro [61]. However, IBI322 has minimal effects on CD47 single positive cells, such as human RBCs. Additionally, IBI322 accumulates in PD-L1^+^ Raji tumors to produce a synergistic effect, resulting in complete tumor regression *in viv*o [61]. Moreover, IBI322 demonstrates minimal destruction of RBCs, and IBI322 is well tolerated in repeated weekly injections, supporting the sufficient therapeutic window in the future clinical use of IBI322 for cancer therapy [61]. Further investigations are required to validate the safety and efficacy of IBI322 for the treatment of hematological malignancies.

### CD47/PD-1 BsAb

CD47/PD-1 BsAb (HX009) is a humanized antibody fusion protein that binds to CD47 and PD-1 concurrently [62]. HX009 considerably diminished solid tumor proliferation in mouse xenograft models [62]. The first-in-human phase I dosage escalation study on HX009 was completed in individuals with advanced solid tumors. HX009 was well tolerated without DLT and exhibited enhanced anticancer efficacy in multiple tumor types [62]. A phase I/II clinical trial is ongoing to evaluate the safety and efficacy of HX009 in the treatment of R/R-lymphoma (NCT05189093). Further investigations of HX009 in hematological malignancies are warranted.

### CD47/CD38 BsAb

CD47 mAb or CD38 mAb is effective for the treatment of MM [92, 113, 184]. Significantly, blocking the CD47/SIRPα signaling pathway enhanced the killing effect of CD38 mAb on MM cells [112]. Based on this, the design of BsAbs targeted both CD47 and CD38 may have a synergistic effect in the treatment of MM. ISB 1442, a fully human BsAb with anti-CD38 and CD47 binding arms, was then developed for the treatment of R/R MM [185]. The CD38 Fab arm of ISB 1442 preferentially drives the binding to CD38^+^ MM cells and achieves a blockade of the proximal CD47 receptor on the same cells through induced binding [185]. The Fc portion of ISB 1442 was engineered to enhance ADCP, ADCC, and CDC. ISB 1442 was highly effective in killing both CD38^hi^ MM cells via CDC and CD38^low^ MM cells via ADCC and ADCP, in comparison to daratumumab in vitro. Significantly, the tumor-killing effect of ADCP, ADCC, and CDC induced by ISB 1442 is more powerful than magrolimab in vitro [185]. Furthermore, there was a twofold increase in MM cell killing with ISB 1442 compared to daratumumab in co-culture assays (macrophages and PBMCs from healthy donors were incubated with MM cells and human serum) [185]. To evaluate the off-target tumor specificity of ISB 1442 in vitro through binding to human RBCs, hemagglutination, and RBC depletion, hemolysis and platelet aggregation were measured in the present study. Compared to magrolimab, ISB 1442 showed no evidence of hemolysis, RBC depletion, or platelet aggregation and significantly reduced human RBC hemagglutination in vitro. In addition, the potency of ISB 1442 was evaluated in vivo using a Raji tumor xenograft model in CB17/SCID mice (which have functional complement, macrophages, and NK cells). Compared to daratumumab, there was a greater inhibition of tumor growth with ISB 1442, and, compared to magrolimab, there was a comparable reduction in tumor size. Collectively, ISB 1442 is anticipated to be a safe and effective approach for the treatment of MM. Therefore, further clinical studies on ISB 1442 are required.

### SIRPα/CD70 BsAb

CD70 is expressed by various hematopoietic and epithelial-derived cancer cells and plays a role in promoting tumor cell survival/proliferation [186]. Phase I clinical trial results have shown that the combination of CD70 mAb (ARGX-110) and azacitidine in patients with AML resulted in an ORR of 92% [187], while single-agent ARGX-110 in advanced cutaneous T-cell lymphoma showed an ORR of 23% [188]. Thereafter, researchers developed a bispecific anti-CD70/SIRPα antibody [189]. In comparison to CD70 mAb + SIRPα mAb treatment, CD70/SIRPα BsAb had a greater ability to phagocytose CD70-expressing NHL and renal cell carcinomas in vitro, but no apparent differences were observed in vivo [189]. Further studies are required to investigate the efficacies of CD70/SIRPα BsAb in CD70^hi^ hematological malignancies.

### SIRPα/CD123 BsAb

CD123 is highly expressed on AML blasts and leukemic stem cells (LSCs) and demonstrates only a moderate expression on normal HSCs, suggesting that CD123 is a promising target antigen [190]. SIRPα receptors on macrophages interact with CD47 to inhibit phagocytosis. SIRPα/CD123 BsAb was designed by *Siret Tahk* et al. based on these interactions [191]*.* Their results demonstrate that SIRPα/CD123 BsAb yields strong anticancer activity against AML in vitro and in vivo via enhanced NK cell-dependent ADCC and macrophage-mediated ADCP effects. SIRPα/CD123 BsAb also established its safety by demonstrating low CD47-related on-target off-leukemia toxicity. Preclinical and clinical testing of the SIRPα/CD123 BsAb is required.

#### CD47/CD3 BsAb

Significant progress has been made in research in Bispecific T cell engagers (BiTE) [192] and anti-CD47/SIRPα antibodies [30, 93] in the treatment of hematological malignancies. Based on this theory, a novel compound, pegylated anti-CD3 x anti-CD47, was developed [193]. The CD47/CD3 BsAb can work via dual mechanisms of both a BiTE and an innate immune checkpoint blockade. The safety of this CD47/CD3 BsAb has been evaluated, and a study has shown that CD47/CD3 BsAb does not induce hemolysis and has slightly elevated T-cell apoptosis [193]. In addition, the half-life of CD47/CD3 BsAb in C57BL/6 mice was 18.4 h for a single dose of 1 mg/kg [193]. Current preclinical studies are ongoing, and clinical studies in hematological malignancies would also be useful.

**Fig. 5:**
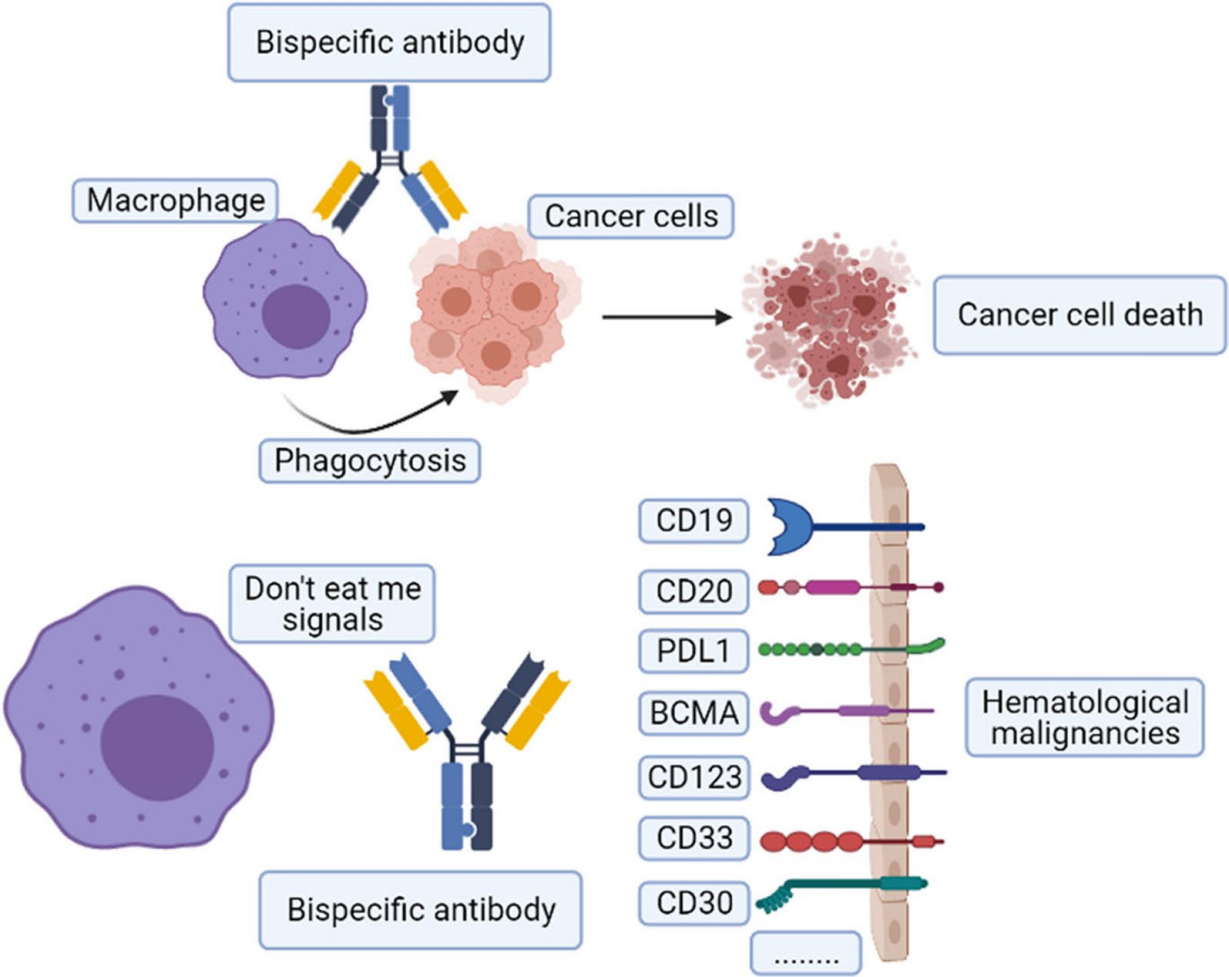
Current and potential CD47/SIRPα-targeted bispecific antibodies (BsAbs) in hematological malignancies: hematological malignancies can be treated with BsAbs that bind “don’t eat me” signal molecules expressed by macrophages and markers specific to tumor cells

Based on this research, CD47/SIRPα-targeted BsAbs are potential treatment options for hematological malignancies. Figure 5 illustrates the target selections of effector cells with BsAbs for cancer therapy. The selected ongoing clinical trials of CD47/SIRPα-targeted BsAbs in hematological malignancies are presented in Table 2. Furthermore, CD47/SIRPα-targeted BsAbs are on the frontiers of novel antibody development [192]. More CD47/SIRPα-targeted BsAbs for hematological malignancies are on the way. Research on tri- or even tetra-specific antibodies has provided a conceptual breakthrough for a new cancer therapy [194], and studies of CD47/SIRPα-targeted tri- or tetra-specific antibodies for hematological malignancies are additionally required.

**Table 2:** Clinical trials of CD47/SIRPα-targeted bispecific antibodies in hematological malignancies

Name	Initial time of clinical studies	phase	Type of tumors	ClinicalTrials.gov Identifier	Outcome measures
TG-1801 (NI-1701)	2021.4	Phase IB	B-cell lymphoma or CLL	NCT04806035	To determine the recommended phase II dose (RP2D), ORR
IMM0306	2021.8	Phase I	R/R CD20-positive B-NHL	NCT04746131	Maximum Tolerated Dose (MTD) of IMM0306 as measured by incidence of DLTs (Dose Limiting Toxicity)
IBI-322	2021.5	Phase I	Hematological malignancies	NCT04795128	treatment related AEs, ORR
IBI-322	2021.12	Phase IA/IB	Myeloid Tumor	NCT05148442	Treatment related AEs, ORR
DSP107	2021.9	Phase IB/II	MDS, AML and T-cell lymphoproliferative disease	NCT04937166	Treatment related AEs, Dose Limiting Toxicities (DLT), ORR, MRD
HX009	2021.12	Phase I/II	R/R-lymphoma	NCT05189093	Treatment related AEs, ORR, PFS, DOR, DCR, PK/PC
JMT601 (CPO107)	2021.12	Phase I/II	Advanced CD20-positive NHL	NCT04853329	To determine the recommended single-agent CPO107 RP2D, safety, efficacy
SG2501	2022.4	Phase IA/IB	R/R hematological malignancies and lymphoma	NCT05293912	To evaluate the safety, tolerability, pharmacokinetics, pharmacodynamics, immunogenicity, and preliminary efficacy

#### Challenges of CD47/SIRPα-targeted BsAbs

The challenges of therapeutic BsAbs should also be noted: (1) The first obstacle in developing BsAbs is the preparation platform technology. BsAbs are broadly divided into two groups: IgG-like (with the Fc region) and non-IgG-like (without the Fc region) [117]. The advantages and disadvantages of these two technology platforms are summarized in Table 3. (2) Appropriate preclinical evaluation models are another challenge in developing BsAbs. Conventional animal models do not have the target-binding characteristics that humans do. For example, the expression of the target, binding ability, pharmacological effects, and upstream and downstream signals of humanized animal models differ from those of humans, making it difficult to correctly evaluate the target design rationality, pharmacological effects, and toxicological effects in preclinical evaluation models. Current preclinical evaluation models usually use alternative molecular, humanized animal models and minimum anticipated biological effect level (MABEL) methods. Although these models can reflect the mechanisms, pharmacological effects, toxic targets, and toxicity phenomena of BsAbs to some extent, the effective dose, toxic dose, and toxic side effects obtained during preclinical evaluation generally cannot be directly converted to clinical doses due to differences in the species’ target expression, binding capacity, pharmacological effects, drug pharmacokinetics and pharmacodynamics (PK/PD) and species type. Furthermore, the results from animal experiments may even be somewhat misleading, increasing the risk of clinical study failure. In light of these issues, more appropriate models and methods for preclinical evaluation of BsAbs need to be developed. Additionally, restrictions on testing in non-human primates, such as chimpanzees, need to be relaxed so that preclinical evaluation of BsAbs can be better performed. (3) Finally, due to the general lack of detailed basic studies on the synergistic effects between target antigens, the selection of target antigens for the development of BsAbs remains a significant challenge in clinical applications. Therefore, it is pivotal to extensively test the safety and effectiveness of BsAbs both in vitro and in vivo before applying them clinically.

**Table 3:** General characteristics of the bispecific antibody technology platforms

	With Fc fragments	Without Fc fragments
Advantages	CMC: Better solubility Better stability Therapeutic effect: Including ADCC and CDC effects, multiple mechanisms to enhance cancer-killing effect Longer half-life	CMC: Easy to produce High productivity Small molecular weight Therapeutic effect: The therapeutic effect is only through antigen binding, with low immunogenicity and fewer side effects
Disadvantages	Some structural CMCs are more complex, mostly with higher aggregation, mismatch and low purification rates If the molecular weight is too large, the permeability to the tumor tissue will be poor	Need to develop specific purification technology routes Shorter half-life, higher dosing frequency and poor patient tolerance

## Reprogramming pro-tumor macrophages as anti-tumor macrophages

M1 macrophages display anti-tumor effects, whereas M2 macrophages demonstrate pro-tumor effects [63]. The polarization of tumor-enhancing M2 macrophages to anticancer M1 macrophages reverses the immunosuppression of the TME [63]. Therefore, reprogramming M2 macrophages into M1 macrophages has emerged as a possible approach to cancer immunotherapy. Numerous reprogramming mechanisms have been reported [63, 64, 195–197]. The following are involved in these reprogramming mechanisms: anti-macrophage receptors with collagenous structure (anti-MARCO) therapy, toll-like receptor (TLR) agonists, T cell immunoglobulin, and mucin domain containing 4 (Tim-4) blockades.

### Anti‑MARCO therapy

MARCO constitutes a pattern recognition receptor that is a member of the scavenger receptor family of class A receptors [198]. MARCO is mostly expressed by TAMs and has been correlated with a poor prognosis in many cancers [199, 200]. In preclinical studies, anti-MARCO antibodies have been reported to inhibit tumor growth and metastasis in 4T1 mammary carcinoma and B16 melanoma mouse models [201]. The anti-tumor activity of anti-MARCO is dependent on the binding of the Fc subunit with its inhibitory Fc receptor, FcγRIIB, similar to anti-CD40 antibody-mediated reprogramming [202]*.* In 2020, *Silke Eisinger* et al. demonstrated a novel mechanism targeting MARCO on tumor macrophages and altering their polarization. In turn, NK cells were activated to kill tumor cells [200]. Anti-MARCO treatment often works in combination with T cell-targeted checkpoint therapies. This finding was confirmed in human-based experiments, wherein a new specific antibody targeting human macrophages could initiate NK cell killing and thus support the use of combinatory treatments in cancer therapy.

In summary, these studies highlight the potential of antibody-mediated macrophage reprogramming using macrophage-associated targets. These studies also stress the significance of the correct antibody designs, especially in the Fc region, for future clinical interventions. A recent study from our group exhibited that a subpopulation of macrophages in the AML microenvironment expressed high levels of MARCO, and MARCO^high^ macrophages shared the M2 phenotype [203]. These results suggest that AML patients with high MARCO expression may benefit from anti-MARCO therapy. Furthermore, MARCO expression in hematological malignancies should be examined in order to demonstrate how anti-MARCO treatment might be effective.

### TLR agonists

TLRs are a pathogen sensor family that recognizes bacterial and viral ligands and activates innate immune sensing [204]. TLR activation polarizes macrophages toward a pro-inflammatory phenotype [204]. Recently, *Holly M. Akilesh* et al*.* reported that activation of TLR7 and TLR9 increases the phagocytosis of monocyte-derived macrophages and causes anemia of inflammation [205]. Researchers have used different TLR ligands in various cancer models to analyze their activities during the transformation of TAMs into tumor-killing macrophages [204].

As early as the 1960s, *Vassal *et al*.* reported improved OS in pediatric leukemia patients using the TLR agonist Bacille Calmette-Guérin vaccine, which is currently used for treating bladder cancer patients [206]. The majority of clinical trials using TLR agonists to treat hematological malignancies have focused on TLR3, TLR7/8, and TLR9. Recent investigations have shown that the synchronous application of different TLR agonists may be useful for patients with various TLRs expressed in tumors. *Brenda J. Weigel* et al. performed a phase II clinical study of 852A in R/R hematological cancer patients [207]. The study reported that six patients had AML, five had ALL, four had NHL, one had HL, and one had MM. The mean age of the patients was 41 years (range: 12–71 years), and the median cycle of prior chemotherapy regimens was five (range: 1–14). Of the 17 cases, 13 patients completed all 24 cycles of 852A injections. Grade 3/4 toxicities included dyspnea, myalgia, nausea, malaise, fever, and cough. Patients with clinical responses included one ALL and one AML. However, nine patients showed progressive disease [207].

TACL T2009-008, a phase I clinical study, indicated that GNKG168 treatment was correlated with immunological changes in 23 patients with pediatric leukemia [208]. However, other studies with large sample sizes are required to investigate the effect of changes in disease therapy and the persistence of leukemia remission. Presently, three phase I/II studies are recruiting patients to assess the clinical efficacy of TLR3, TLR4, and TLR9 agonists combined with standard therapies for the treatment of R/R lymphomas, including low-grade B cell lymphomas, T cell lymphomas, and FL (Clinical Trial Identifiers: NCT01976585, NCT03410901, and NCT02927964).

In conclusion, TLR agonists activate macrophages toward the M1 phenotype, resulting in satisfactory preclinical therapeutic effects for hematological malignancies. Future studies are required to gain a comprehensive mechanistic understanding of the role of TLR agonists in hematological malignancies. Additionally, there is a need to examine the influence of TLR signaling on the pathogenesis of hematological malignancies and determine the appropriate clinical utility by conducting extensive cohort studies.

### T cell immunoglobulin and mucin domain containing 4 (Tim-4) blockade

Under normal conditions, Tim-4^+^ macrophages recognize phosphatidylserine (PS) receptors exposed on the apoptotic cell surface or on the nuclei extruded from matured erythroid cells. Tim-4^+^ macrophages then remove these cells, thereby maintaining homeostasis and preventing the development of autoimmune diseases [72, 209]. In 2020, *Houjun Xia* et al. reported that Tim-4^+^ TAMs in the peritoneal cavity of ovarian cancer are embryonic in origin and locally sustained through self-renewal, while Tim-4^−^ TAMs are replenished from circulating monocytes [210]. In the ID8 ovarian cancer mouse model, *Houjun Xia* et al. found that Tim-4^+^ TAMs, but not Tim-4^−^ TAMs, promote the proliferation of cancer in vivo [210]. RNA-sequencing analysis revealed that Tim-4^+^ and Tim-4^−^ TAMs have different functions and phenotypes. The Tim-4^+^ TAMs exhibit higher levels of arginase-1 (ARG1), inhibiting mTORC1 activation and thus promoting mitophagy [210]. Moreover, deficiency of the autophagy element FAK family-interacting protein 200 kDa (FIP200) led to Tim-4^+^ TAM loss, enhanced T cell immune responses, and inhibited the proliferation of the ID8 tumor in vivo [210]. The complement receptor of the immunoglobulin superfamily (CRIg)^+^ TAMs in human ovarian cancer is similar to murine Tim-4^+^ TAMs in terms of transcriptional levels, metabolic effects, and functional roles. Accordingly, targeting CRIg^+^ (Tim-4^+^) TAMs may effectively treat patients with peritoneal metastases from ovarian cancer [210]. *Anders Etzerodt* et al. reported similar results in 2020 [211]. Their findings revealed the metastatic spread of ovarian cancer cells via a distinct population of omental macrophages with CD163^+^ Tim4^+^ on their surface [211]. Using genetic and pharmacological tools, selectively depleting of CD163^+^ Tim4^+^ macrophages could prevent tumor progression and metastatic spread by in the omentum [211]. *María Casanova-Acebes* et al. also reported that the peritoneal TAMs of ovarian cancer significantly promote tumor progression [212]. Additionally, the researchers demonstrated that RXR signaling controls the maintenance of these TAMs and that the deletion of RXR diminishes the accumulation of TAMs in tumors and strongly reduces ovarian tumor progression in mice [212]. In 2021, *Andrew Chow* et al. reported a poor prognosis after PD-1 therapy for metastatic cancer of the pleural and peritoneal cavities [213]. First, they discovered that Tim4^+^ TAMs are positively related to decreased numbers of CD8^+^ T lymphocytes with tumor-reactive characteristics in cancer patients’ pleural effusions and peritoneal ascites. Additionally, they observed that Tim-4^+^ macrophages trap and inhibit CD8^+^ T cell growth. In contrast, Tim-4 inhibition abolishes this sequestration and growth suppression and significantly improves anti-tumor effectiveness in mouse models of anti-PD-1 treatment and adoptive T cell therapy. Thus, these investigations recommend Tim-4 as a TAM target, having demonstrated that blocking Tim-4 enhances anti-PD-1 treatment in preclinical tests.

Tim-4 is highly expressed on the surface of macrophages in hematopoietic-related organs, bone marrow, spleen, lymph node, fetal liver, and so on [72, 209]. Additional study of the functional roles of Tim-4^+^ macrophages in hematological malignancies is required.

## Chimeric antigen receptor-macrophage (CAR-M)

Therapeutic success in targeting tumor-promotional roles in preclinical and clinical cancer treatment investigations suggests that TAMs are attractive immune-targeted cells for monotherapy or combination therapy. Depletion of immunosuppressive TAMs or enhancement of macrophage phagocytosis has been reported in clinical trials on hematological malignancies [100, 157, 214]. However, it is important to note that current macrophage-targeted therapeutic approaches are mechanistically dependent on TAMs. TAMs express activating- and inhibitory-Fc receptors, which can polarize toward either a tumor-promotional or immunosuppressive phenotype [66]. However, the clinical efficacies of monotherapy using these macrophage-targeted therapies are limited due to the lack of available clinical trial data. Clinical trials are necessary to further investigate novel specific macrophage-targeted therapies.

### Preclinical studies of CAR-M

*Meghan A. Morrissey* et al. developed a family of chimeric antigen receptors that can guide macrophages to phagocytose antigen-specific targets, including Raji B cells, CD19, or CD22 positive cells [215]. In their study, three engineering strategies were tested. Macrophages expressing the CAR-P^Megf10^ or CAR-P^FcRɣ^ were insufficient to trigger whole-cell engulfment [215]. Moreover, in conjunction with CD47 blockade led to a 2.5-fold increase of phagocytosis of Raji cells, suggesting CAR-P expression in combination with CD47 or SIRPα blockades has an additive effect [215]. In contrast, CAR-P^tandem^ enhanced the capability of macrophages to take up cancer cells, suggesting that assembling a motif of phagocytic effectors could increase the cancer cell phagocytic activity of CAR-P [66, 216]. This engineered macrophage could be identified as the first reported “CAR-M” in preclinical studies. Furthermore, the macrophages showed antigen-specific killing effects among tumor cells. In 2020, *Michael Klichinsky* et al. established a novel cellular therapy, CAR macrophages (CAR-Ms) [66]. These researchers hypothesized that human macrophages expressing CARs could redirect their phagocytosis function, resulting in an antigen-specific, anticancer therapeutic effect with the potential to induce an adaptive immune response. CAR-Ms led to a sustained pro-inflammatory phenotype and overcame the inherent phagocytosis-correlated resistance of primary human macrophages. In addition, CAR-Ms demonstrated antigen-specific phagocytosis and clearance of tumor cells in vitro [66]. A single transfusion of reprogrammed human CAR-Ms led to a remarkable inhibition of tumor growth and prolonged OS of mice using xenograft mouse models of solid tumor in vivo.

**Fig. 6:**
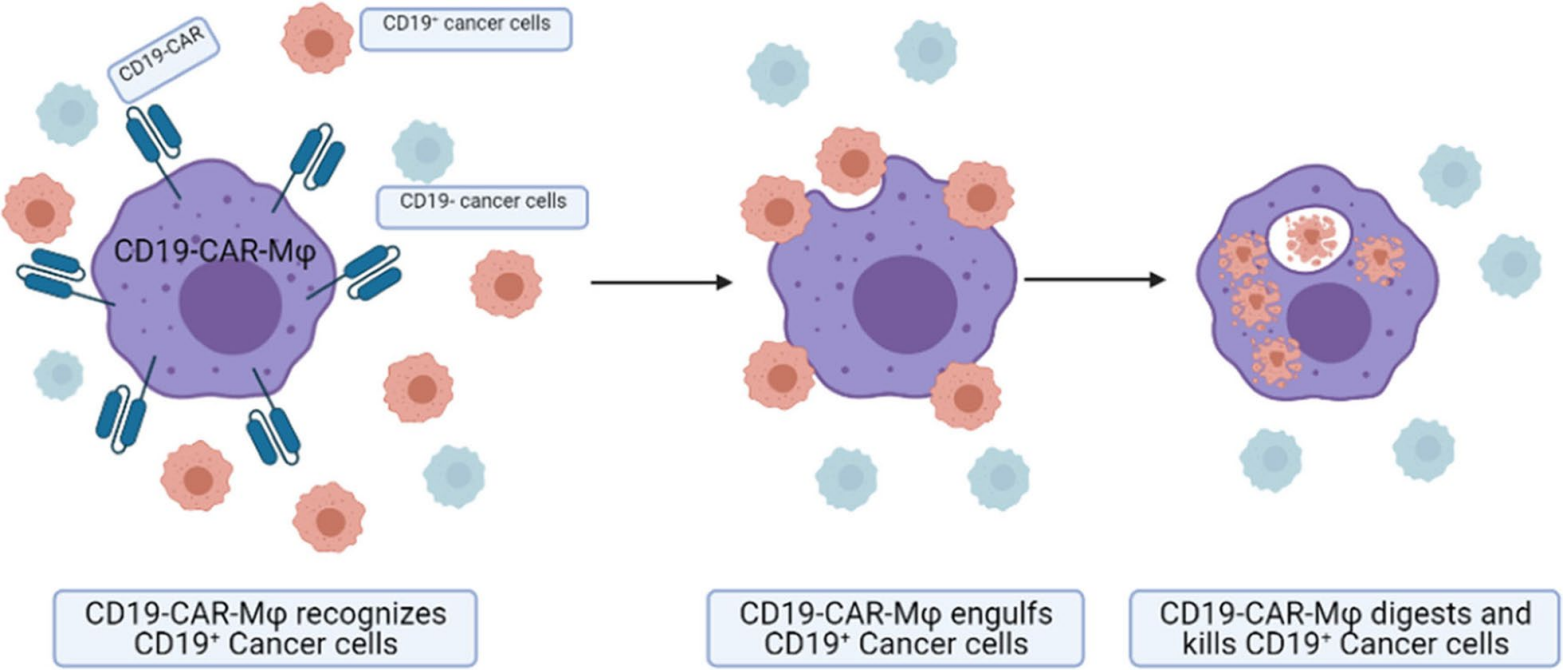
Processes by which CD19 CAR macrophages specifically recognize, phagocytose, digest, and kill CD19^+^ cancer cells: CD19-CAR macrophages can recognize, engulf, digest, and kill CD19^+^ cancer cells but not CD19^−^ cancer cells

Transduced CARs were constructed to define the mechanism of CAR-modulated redirection of macrophage phagocytosis: CAR19ζ, which contains a CD3ζ intracellular domain, and CAR19Δζ, which lacks the CD3ζ intracellular domain. The CD3ζ molecule is homologous with the Fc common γ-chain, FcεRI-γ molecule, and ADCP signaling molecule in macrophages [66]. Therefore, only CAR19ζ was found to phagocytose CD19^+^ cancer cells in an antigen-specific manner, unlike CAR19Δζ and control untransduced (UTD) macrophages (Fig. 6) [66]. In addition, the engulfment of CAR-Ms is considered an active process requiring the polymerization of Syk, actin, and non-muscle myosin-IIA. The phagocytic process is similar to the Fc receptor-mediated ADCP. Transcriptome sequencing data demonstrate that CAR-Ms secrete pro-inflammatory cytokines and chemokines, convert M2 macrophages to M1, upregulate antigen presentation, recruit and present antigens to T cells, and resist the effects of immunosuppressive cytokines [66]. CAR-Ms yield substantially increased antigen-specific anti-tumor effects for hematological malignancies.

However, sourcing and the high cost of CAR-Ms remain a challenge. Immortalized macrophage cell lines are not applicable for clinical use, and bone marrow (BM) and peripheral blood mononuclear cells (PBMC)-derived macrophages cannot be efficiently modified or engineered. There is a need for novel and efficiently engineered macrophages [65]. Recently, induced pluripotent stem cells (iPSCs)-derived macrophages (CAR-iMac) have emerged as a promising source of cellular immunotherapy following the work of *Li Zhang* et al. [65]. They demonstrated that CAR-iMac cells result in antigen-specific functions for macrophages, such as the secretion of pro-inflammatory cytokines, polarization toward the anti-tumor phenotype, engulfment of antigen-specific tumor cells, and *in vivo* anti-tumor cell activity for both hematological malignancies and solid tumors [65]. Hence, this study presents a new source of CAR-M with satisfactory anti-tumor cell activity.

### Clinical studies of CAR-M

Preclinical findings of CAR-M (CT-0508) suggest that this therapy may have the potential to overcome the challenges encountered with T cell therapies in solid tumors [66]. On December 9, 2020, Carisma Therapeutics Inc. began the first clinical study of CAR-M (CT-0508): “A phase I, the first human study of adenoviral transduced autologous macrophages engineered to contain an anti-HER2 chimeric antigen receptor in subjects with HER2-overexpressing solid tumors” (ClinicalTrials.gov Identifier: NCT04660929). This clinical trial represents an important milestone in the development of gene-based therapies because it marks the first reported use of CAR-Ms in a clinical study. The study focused on solid tumors in patients with recurrent or metastatic human epidermal growth factor receptor 2 (HER2)-overexpression for cancers without any approved HER2-targeted therapies or patients who did not respond to treatment. On March 18, 2021, Carisma Therapeutics Inc. announced that the first patient had received the first dose of CT-0508, HER2-targeted CAR-M, in the phase I multicenter clinical trial. However, more time is necessary to investigate the clinical efficacy of this CAR-M.

The success of CAR-T cells in hematological malignancies suggests that CAR-M cellular therapy may also yield promising results in hematological malignancies. Indeed, IMAGINE (ClinicalTrials.gov Identifier: NCT05138458) is a multicenter, open-label, dose escalation and dose cohort expansion phase I/II clinical trial for evaluating CD5-targeted CAR-M (MT-101) treatment in patients with R/R-PTCL. This study was sponsored by Myeloid Therapeutics on December 15, 2021. On May 11, 2022, they completed the first patient dosing of MT-101 in R/R-PTCL. This patient did not exhibit cytokine release syndrome (CRS) and no apparent cytotoxicity. However, more time is needed to investigate the safety and clinical efficacy of MT-101.

Several diverse CAR-Ms have been evaluated for treating both hematological malignancies and solid tumors in preclinical studies and clinical studies [217, 218] (Table 4). The target antigen included CD5, CD19, CD22, HER2, CCR7, and ALK. More target antigens must be developed in the future to explore their therapeutic role in hematological malignancies.

**Table 4:** Characteristics and anti-tumor effects of CAR macrophage

Source of CAR-M	Target antigens	CAR Structure	Generation	Stage	Preclinical results	Refs
J774A.1	CD19 and CD22	CD19 + Megf10 CD19 + FcRV CD19 + FcRV + PI3K	1st generation	Preclinical evaluation	CD3ζ, FcRγ and Megf10 intracellular domains demonstrate similar phagocytic activity in vitro and PI3K recruitment domain enhances phagocytosis of whole tumor cells CD47 mAb enhances the phagocytic activity of CAR-M in vitro;	212
iPSCs	CD19	CD19 + 4–1BB + CD3ζ	2nd generation	Preclinical evaluation	CAR-iMACs demonstrate pro-inflammatory/anti-tumor state in vitro CAR-iMACs can expand, persist and exert anti-tumor activities in vivo	64
Raw264.7	HER2	HER2 + CD147	1st generation	Preclinical evaluation	CAR-147 induces MMP expression in vitro and in vivo, but does not enhance phagocytosis of tumor cells CAR-147 lowers collagen content, increases CD3^+^ T cell infiltration and inhibits the proliferation of tumor	214
Raw264.7	CCR7	CCL19 + TLR2, TLR4 + TLR6 + MerTk + 4-1BB + CD3ζ	2nd generation	Preclinical evaluation	In vitro, MerTK (CAR-M) kills and phagocytoses cells more efficiently than CAR-M that has other intracellular domains CAR-M (MerTK) inhibits the progression of tumors, prolongs survival time, and inhibits tumor metastasis in mice with minimal toxicity Cancer cells treated with CAR-M (MerTK) exhibit significant CD3^+^ T cell infiltration, a decrease of PD-L1-positive cells, as well as an increase in pro-inflammatory cytokines	66
Monocytes	HER2	HER2 + CD3ζ	2nd generation	Phase I Ongoing clinical trials (CT0508) against HER2^+^ solid tumors	Injection of adenoviral vector leads to M1-like macrophages and pro-inflammatory microenvironment in the tumor An adenovirus-transduced CAR macrophage is capable of cross-presenting tumor-derived antigens and activating T lymphocytes more effectively An adenovirus-transduced CAR macrophage significantly prolongs survival time and decreases metastasis in mice harboring tumors;	65
Human THP-1	CD19	CD19 + CD3ζ	2nd generation	Preclinical evaluation	CAR-M enhances antigen-dependent phagocytosis in vitro	65
Murine BMDM	ALK	ALK + CD8 Hinge + CD28 TM + CD28 CSD + CD3ζ + IFN-γ	3rd generation	Preclinical evaluation	CAR-M enhances antigen-dependent phagocytosis CAR-M induces M1 polarization, upregulates antigen presentation, increases T cell activations, and reduces tumor burden	215
Monocytes	CD5	Un	mRNA engineered	Phase I Ongoing clinical trials (MT-101) against PTCL	Un	Un

*Un* Unknown

### Strengths and challenges of CAR-M

In conclusion, CAR-Ms display antigen-specific anti-tumor activities in preclinical studies both in vitro and in vivo [219]. This novel cellular immunotherapy has clear potential for treating both hematological malignancies and solid tumors [220]. The strengths of CAR-M include the following points: 1) CAR-M can infiltrate tumor tissues considerably, reduce the proportion of TAMs, affect the phenotype of TAMs, and produce a positive effect on tumor treatment. 2) In addition to its role in tumor cell phagocytosis, CAR-M can promote antigen presentation and enhance T cell killing. 3) CAR-M shows little “On-Target, Off-Tumor Toxicity.”

The challenges associated with CAR-M’s wider applications in various cancer types should also be considered in future studies. The challenges include the following points: 1) The safety and efficacy of CAR-M for humans remain to be proved via clinical trials. 2) Reliable cell sources and expansion are necessary for the clinical application of CAR-M. 3) Currently, CAR-M mostly uses viral transfection, which may induce insertional mutations; however, CRISPR/Cas9 offers a solution. 4) The underlying mechanisms of CAR-M resistance need to be investigated. 5) The complex immune microenvironment should also be considered when applying CAR-M therapy. Thus, rational selection of existing therapy in combination with CAR-Ms may have a synergistic effect on anti-tumor responses.

## Conclusion and future perspectives

Recently, understanding the correlation between the immune state and tumor growth has led to the development of several immunotherapies. CTLA-4 and PD-1/PD-L1 blockades have been used in the treatment of several cancer types. However, the ORR of these blockades remains low, highlighting the fact that other immune escape mechanisms exist in the highly heterogeneous TME. Phagocytosis by macrophages and subsequent immune recognition of tumor cells has been increasingly recognized as being governed by multiple “eat me” and “don’t eat me” signals that may have the potential to generate optimal anti-tumor responses. There are four main cross talks between macrophages and tumor cells that have been identified as “don’t eat me” signals. The CD47/SIRPα axis was the first phagocytosis checkpoint discovered in cancer, and afterward, other phagocytosis checkpoints (PD-1/PD-L1, MHC-I/LILRB1 axis, and CD24/SIGLEC-10 axis) have been identified. Often, these phagocytosis checkpoints are not potent enough to be used alone but can potentiate the clinical effects of existing therapy. Additional clinical trials are ongoing to determine these strategies’ clinical efficacy and AEs. Nevertheless, future studies should pay more attention to the dual activities of blocking “don’t eat me” and activating “eat me” signal pathways during the process of the anti-tumor treatment. The deletion of macrophages has shown potential therapeutic effects for hematological malignancies; however, their combination with phagocytosis checkpoint inhibitors should be avoided. CD47/SIRPα-targeted BsAbs have shown promising preclinical results and represent promising approaches to immunotherapy. However, they are still in the beginning stages of clinical development in hematological malignancies. Currently, several BsAbs are being examined in clinical trials for use in cancer therapy. Reprogramming pro-tumor macrophages to anti-tumor macrophages via anti-MARCO therapy, TLR agonist, and Tim-4 blockade is also worth examining in future clinical studies. Genetically engineered CAR-M increases antigen-specific phagocytoses and tumor clearance according to preclinical results. None can predict now how CAR-Ms will be used in the future treatment of hematological malignancies, but the results of clinical trials are anticipated. Furthermore, it is necessary to identify more targetable surface antigens, which are highly, specifically, and homogeneously expressed by all blood cancer cells.

Moreover, certain aspects of targeting macrophages have not been fully investigated in hematological malignancies: 1) Mechanistically, the cross talk between phagocytosis regulators and the professional phagocytes in modulating tumor cell clearance during tumorigenesis and among distinct types of blood cancers must be elucidated. 2) From a clinical perspective, incorporating phagocytosis checkpoint blockades into current cancer therapies should be considered. 3) The discovery of new and specific tumor and immune cell targets is also urgently needed to increase efficacy and reduce the AEs of CD47/SIRPα-targeted BsAbs and CD47/SIRPα-targeted ADCs. And studies of CD47/SIRPα-targeted tri- or tetra-specific antibodies are also needed for hematological malignancies. 4) CAR-T cell therapy in combination with macrophage-targeted treatment also deserves further study in hematological malignancies. 5) More clinical trials of CAR-M, either alone or in combination with existing therapy in patients with hematological malignancies, are also required to evaluate the safety and efficacy of CAR-M strategies. 6) Striking a balance between the potency and toxicity of macrophage-targeted therapies is important. 7) Compared with adaptive immune checkpoint inhibitors, innate immune checkpoint inhibitors are less specific and more prone to normal tissue damage. Therefore, future advances in the AEs screening should also be defined. Despite these challenges, macrophage-targeted therapies remain vital and promising tools in the fight against blood cancer.

## Data Availability

Not applicable.

## Abbreviations

ADC: Antibody–drug conjugate
ADCC: Antibody-dependent cellular cytotoxicity
ADCP: Antibody-dependent cellular phagocytosis
AE: Adverse effects
ALL: Acute lymphoblastic leukemia
AML: Acute myeloid leukemia
ARG1: Arginase-1
ASH: American Society of Hematology
AZA: Azacytidine
β_2_-M: β_2_-Microglobulin
BiTE: Bispecific T cell engagers
BM: Bone marrow
BsAbs: Bispecific antibodies
CAILS: Composite assessment of index lesion severity
CAR: Chimeric antigen receptor
CDC: Complement-dependent cytotoxicity
CR: Complete remission
CRS: Cytokine release syndrome
CTCL: Cutaneous T-cell lymphoma
CTLA-4: Cytotoxic T-lymphocyte antigen 4
DCR: Disease control rate
DCs: Dendritic cells
DLBCL: Diffuse large B cell lymphoma
DLT: Dose-limiting toxicity
DOR: Duration of response
EBI: Erythroblastic island
FACS: Fluorescence-activated cell sorting
FL: Follicular lymphoma
HL: Hodgkin's lymphoma
HSCs: Hematopoietic stem cells
iPSCs: Induced pluripotent stem cells
LILR: Leukocyte immunoglobulin-like receptor
MABEL: Minimum anticipated biological effect level
MARCO: Macrophage receptor with collagenous structure
MCL: Mantle cell lymphoma
MDS: Myelodysplastic syndrome
MHC-I: Major histocompatibility complex-I
NHL: Non-Hodgkin Lymphoma
MDSCs: Myeloid-derived stem cells
MHC-I: Major histocompatibility complex
MM: Multiple myeloma
MTD: Maximum tolerated dose
MZL: Marginal zone lymphoma
ORR: Objective response rate
OS: Overall survival time
PBMC: Peripheral blood mononuclear cells
PBO: Placebo
PD-1: Programmed cell death protein 1
PD-L1: Programmed death-ligand 1
PFS: Progression-free survival
PR: Partial responses
PS: Phosphatidylserine
PTCL: Peripheral T-cell lymphoma
RBCs: Red blood cells
RFS: Recurrence-free survival
RPMs: Red pulp macrophages
Sc-RNA-seq: Single cell RNA-sequencing
SD: Stable disease
SIRPα: Signal regulatory protein α
SIGLEC-10: Sialic-acid-binding Ig-like lectin 10
SLAMF7: Signaling lymphocytic activation molecule family member 7
TAM: Tumor-associated macrophage
TCR: T cell receptor
TEAEs: Treatment-emergent adverse events
TILs: Tumor-infiltrating lymphocytes
Tim-4: T cell immunoglobulin and mucin domain containing 4
TLR: Toll-like receptor
TME: Tumor microenvironment
TNBC: Triple-negative breast cancer
TRM: Tissue resident macrophage
TTP: Time to progression
